# Calcia magnesia alumino silicate (CMAS) corrosion attack on thermally sprayed thermal barrier coatings: a comprehensive review

**DOI:** 10.1038/s41529-024-00462-w

**Published:** 2024-04-25

**Authors:** Rakesh Bhaskaran Nair, Dermot Brabazon

**Affiliations:** 1 I-Form, the SFI Research Centre for Advanced Manufacturing, Dublin City University, Dublin, Ireland; 2 School of Mechanical & Manufacturing Engineering, Dublin City University, Dublin, Ireland; 3 Advanced Processing Technology Research Centre, Dublin City University, Dublin, Ireland

**Keywords:** Environmental impact, Scientific community

## Abstract

Calcia-Magnesia-Alumino Silicate (CMAS) is a form of molten siliceous residue generated at elevated temperatures within aeroengines. CMAS adheres to the surface of thermal barrier coatings (TBCs) and has the potential to cause significant damage to engine components, resulting in TBC failures. The aviation industry has long recognized CMAS as a substantial threat to aircraft engines, and this threat persists today. A substantial amount of research has been carried out, primarily focusing on gaining a fundamental understanding of the degradation mechanism of traditional TBCs manufactured using air plasma spraying (APS) and electron beam physical vapor deposition (EB-PVD) technologies after CMAS attack. A thorough understanding of why CMAS forms, its role in causing severe spallation, and how to prevent it is of significant concern both academically and industrially. This review article provides a detailed examination of the chemistry of CMAS and the resulting degradation mechanisms that the TBC may encounter throughout the aeroengine service life. This article also explores recent research, incorporating case studies, on the impact of CMAS attack on the resulting chemical and structural modifications of the ceramic topcoats. Current strategies designed to mitigate CMAS infiltration and perspectives for enhanced mitigation are discussed.

## Introduction

The blades and vanes in aerospace gas turbine engines (GTEs) have been almost exclusively fabricated from nickel-based superalloys due to their remarkable resistance to creep and high-temperature strength^1^. However, in modern aircraft, the turbine inlet temperature (TIT) exceeds 1300 °C in order to attain higher thermodynamic cycle efficiencies, subjecting nickel superalloys to the most severe conditions of temperature and stress compared to any other component within the GTE. The ability of nickel superalloys used in GTEs to endure high temperatures has a direct impact on their advanced operational capabilities. For instance, nickel-based superalloys experience a loss of strength beyond the operating temperature of 1000 °C, leading to adverse effects on GTE operating conditions, increased fuel consumption, and reduced engine cycle efficiency^1–3^. Aircraft manufacturers, in their pursuit of enhancing engine efficiency with reduced emissions, have incorporated an insulative layer to protect the components from catastrophic failure. This insulative layer provides thermal insulation to safeguard the superalloy components, thereby improving the durability of engine components. The insulating layer is commonly known as the thermal barrier coatings (TBCs). The TBCs consist of thermally insulating materials with substantial thickness and durability, enabling them to withstand significant temperature differences between the load-bearing alloy and the surface of the coating^2^. With the advancement of TBC systems, the GTE of aircrafts is continuously aiming for higher efficiency by pushing the TIT above 1400 °C, with future targets exceeding 1600 °C.

**Fig. 1:**
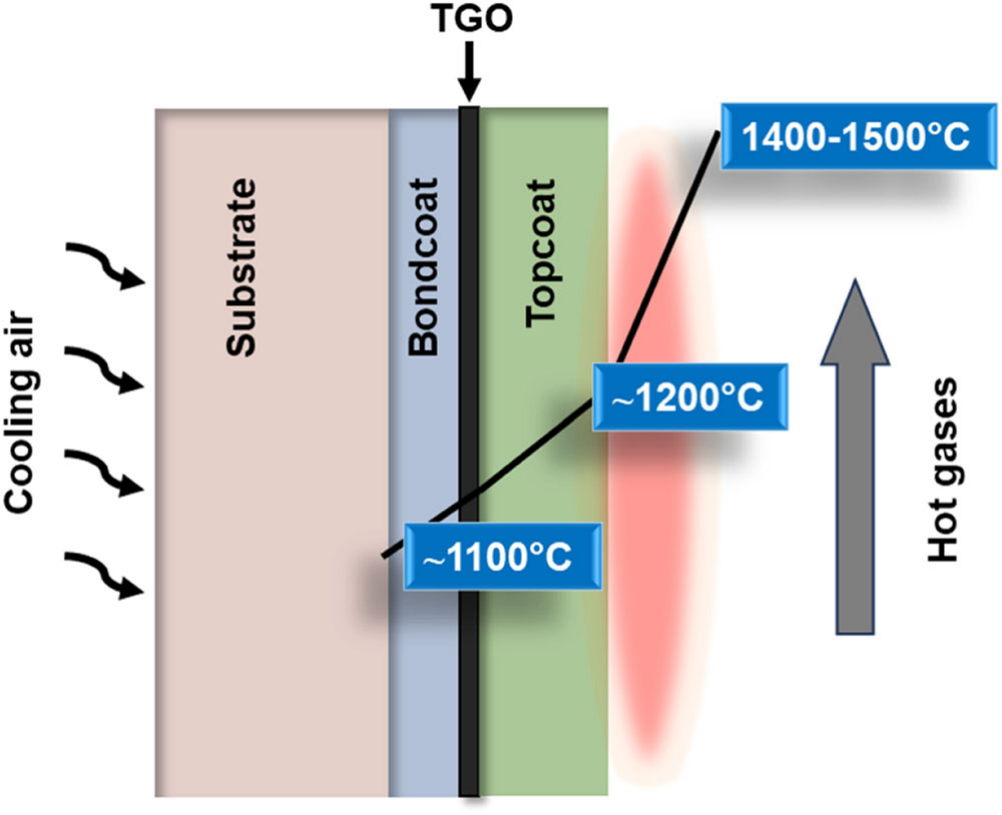
Thermal barrier coating is applied to the surfaces to to reduce heat transfer and to improve durability of the component. Typical temperatures in the combustion gas and thermal barrier coating.

TBCs mainly consist of two layers, a ceramic insulative layer as a topcoat and a layer with bondcoat between the ceramic layer and superalloy substrate, as shown in Fig. 1. The purpose of the metallic bondcoat is threefold: firstly, to safeguard the underlying superalloy substrate from oxidation, secondly, to mitigate thermal mismatch between the topcoat and substrate, and finally, to inhibit the interdiffusion of elements between the substrate and bondcoat. During operations, a reaction byproduct layer referred to as the “thermally grown oxide (TGO)” (typical thickness ranges from 2 µm to 8 µm) develops at the interface between topcoat and bondcoat^2–4^. Ceramic topcoat lacks the ability to protect against oxygen infiltration, rendering it transparent to oxygen owing to the porous structure, hence TGO layer serves as a protective barrier against oxidation and hot corrosion. NiCoCrAly is a preferred choice of bondcoat material because of the stable TGO scale formation (i.e., alumina scale, *α*-Al_2_O_3_)^5,6^. While the aim of reaching the TIT higher (>1500 °C) for aeroengines for engine efficiency improvement, researchers are now focusing on next-generation bondcoats, specifically high-entropy alloys (HEAs)^7^. Conversely, the careful selection of the ceramic topcoat is crucial, as it needs to fulfill essential requirements. These include low thermal conductivity, high thermal shock resistance, resistance to particle erosion, high melting point, high coefficient of thermal expansion (CTE), and good fracture toughness^8^. Zirconia has been examined as a promising candidate owing to its low thermal conductivity compared to those of other ceramic materials^9^. Nevertheless, the pure zirconia undergoes phase transition at higher temperature, hence, the risk of cracking remains^3,10^. During the late 1970s, researchers made a significant discovery that involved adding small amounts of yttria as a dopant to stabilize zirconia. This finding sparked extensive investigation and utilization of yttria-stabilized zirconia in various applications. The selection of 7–8 wt.% yttria-stabilized zirconia (8YSZ) as the preferred material for the topcoat application is attributed to its desirable characteristics compared to pure zirconia. These include low thermal conductivity (~1 W/m^2^ K)^10,11^, high melting point (2680 °C)^10,12^, and a high CTE of 11 × 10^−6^/K at approximately 1000 °C^10^.

**Fig. 2:**
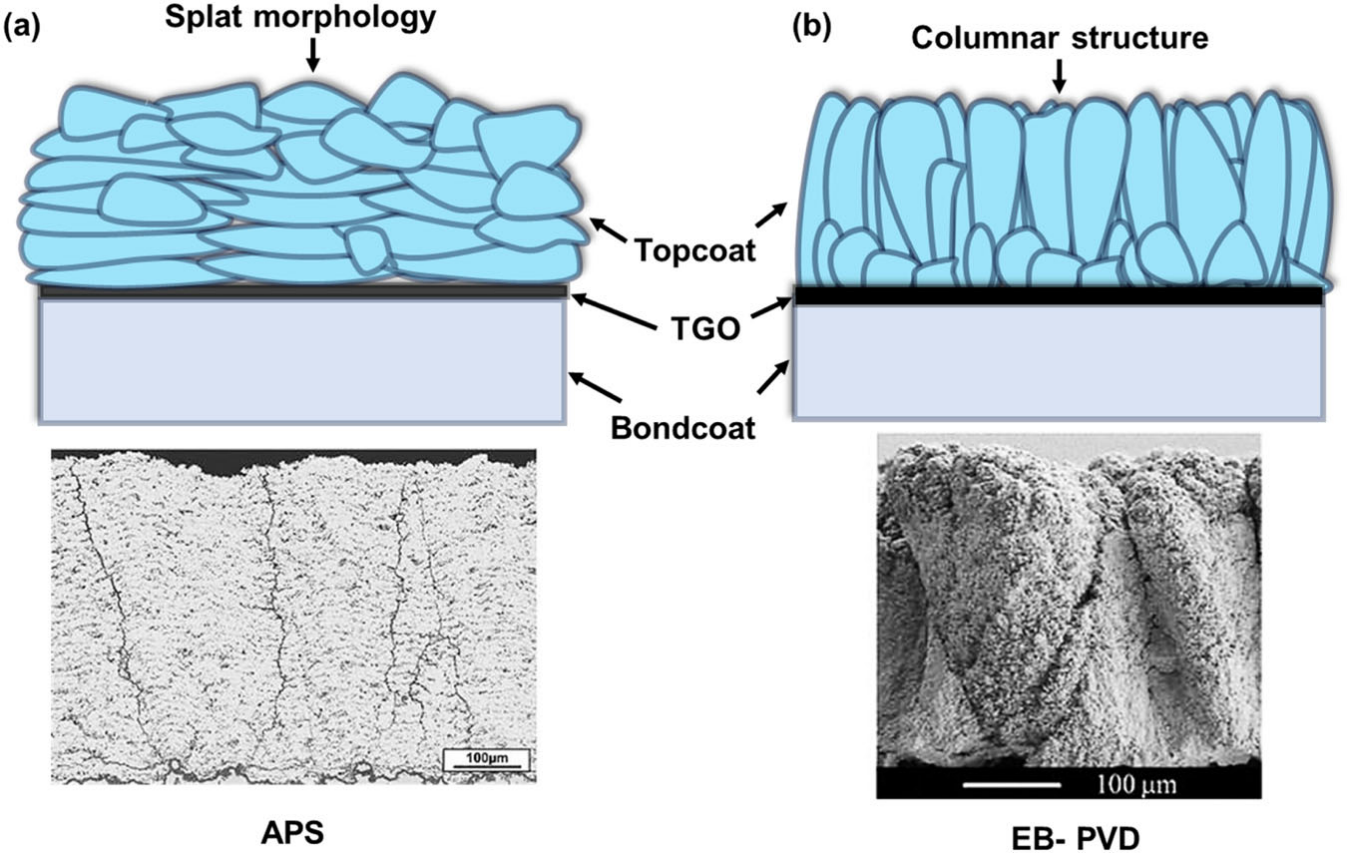
Comparing deposited layer morphologies in thermal barrier coatings via APS and EB-PVD techniques. Schematic illustration of different deposit layer morphology produced by **a** APS and **b** EB-PVD technology in the TBCs and corresponding backscattered SEM images are also provided for better comparison^108^. EB-PVD provides a coating with a high strain tolerance columnar structure, whilst a splat-type lamellar microstructure is produced through conventional air plasma spray technology.

Generally, atmospheric plasma spraying (APS) and electron beam physical vapor deposition (EB-PVD) are employed as deposition techniques for fabricating TBCs resulting in different deposit layer structures, see Fig. 2. The EB-PVD technique is commonly employed to produce TBCs with a columnar structure (Fig. 2b) characterized by multi-scale porosity (porosity ranges between 10 and 20%)^13–15^. This structure serves to reduce thermal conductivity while simultaneously enhancing the coating’s strain tolerance capability^13,15^. On the other hand, APS involves the utilization of a plasma torch comprising a cathode and a nozzle-shaped anode. This configuration generates an electric arc discharge through the ionization of a gas mixture, typically argon and hydrogen^16–18^. Within the plasma torch, the gas mixture is transformed into a high-temperature plasma flame. The plasma atmosphere reaches temperatures ranging from 3000 to 15,000 K, ensuring the melting of particles. This melting is facilitated by the intense thermal energy provided by the plasma flame. A continuous stream of pressurized gas generates plasma, causing the feedstock to transform into countless molten droplets with velocities between 80 and 300 m/s. The molten droplets are accelerated towards the substrate, which impact at high velocity and subsequently solidify into flattened particles known as “splats”, see Fig. 2a. During the cooling process, the formation of intra-splat cracks occurs as a result of splat shrinkage. The porous and crack-like voids structure enhances the strain tolerance of the ceramic topcoat. In addition, the porous structure, which is crucial in order to provide a lower thermal conductivity, also enables the efficient transport of oxygen and other reactive species through the TBCs. The TBC systems have been an active research area for many decades with many review papers previously published related to the development of TBCs^18–20^.

**Fig. 3:**
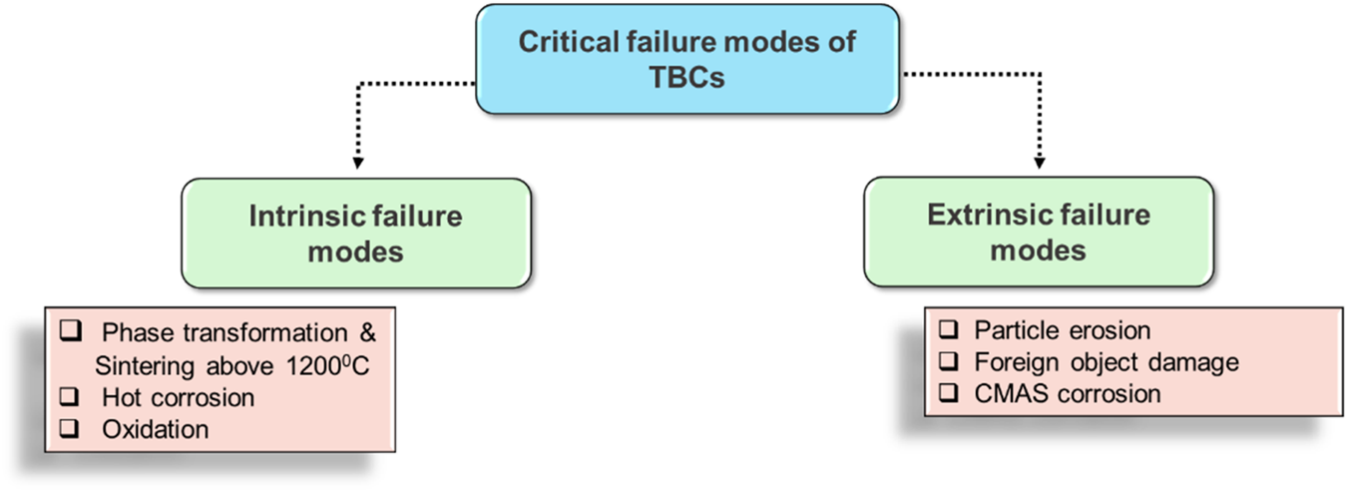
Critical failure modes and factors influencing these for TBC systems.

Despite the potential for enhancing GTE operational efficiency, the traditional yttria-stabilized zirconia TBC systems are susceptible to failure and classified into two categories, intrinsic and extrinsic failure modes, as depicted in Fig. 3. Examples of intrinsic failure mode include flaw formation due to phase transformation or an excessive thermal gradient generated during sintering. In general, the maximum operating temperature of 8YSZ is limited to 1200 °C for long-term operations. According to the study conducted by Brandon and Taylor^21^, while operating at temperature beyond 1200 °C, the non-transformable tetragonal-*t*’ phase of 7–8 wt.% YSZ gradually decomposes into the regular tetragonal-*t* phase (with low yttria content of 0–4 wt.% YSZ) and the cubic-*c* phase (with high yttria content of 13–20 wt.% YSZ), which are the equilibrium phases at high temperatures. When the material is cooled to room temperature, the tetragonal-*t* phase can undergo a phase transformation into the monoclinic-*m* phase, resulting in a volume increase of ~3–5%^21^. Since ceramic materials exhibit minimal plasticity at low temperatures, the stresses arising from this phase transformation tend to be relieved through microcracking, leading to a loss of mechanical strength and ultimately failure of the YSZ coating. In addition to that, densification of microstructure takes place for the 8YSZ-based TBC at elevated temperature (>1200 °C), leading to an increase in thermal conductivity, which can cause early failure of the TBC.

Extrinsic failure modes usually involve TBC damage due to erosion resulting from environmental particulates (typically fine particulates <75 μm) and foreign object damage^22^. The fine particulates typically come from airport runaways, dust sandstorms, and volcanic ash, wherein, the level of particulate concentrations are relatively higher, typically ranges between 350 µg/m^3^ and 13,000 µg/m^3^ ^23^. These fine particles enter the low-pressure compressor region and eventually reach the combustion chamber, which is characterized by high TIT. During high-temperature operating conditions, these fine environmental particulates melt, decompose, and adhere to the components, leading to structural degradation and changes in the chemistry of TBCs. The fine particulates primarily consist of compounds composed of calcia-magnesia-alumino silicate (CMAS). Consequently, the deterioration resulting from these particles is commonly known as “CMAS attack”. The primary focus of this review article is to present the current understanding of the complexities involved in CMAS attack on TBC systems deposited by means of thermal spraying.

## Brief historical overview of CMAS on engine components

In the 1990s Black Hawk helicopters were found to have experienced substantial engine performance deterioration from the presence of desert sand (kind of a glassy deposits) on the leading edge of the high-pressure turbine vanes^23^. The chemistry of the sand analysis revealed the occurrence of calcium-aluminum-silicate glass, low quartz, carbonates (dolomite and calcite), and minor traces of NaCl. The high-temperature regions of the helicopter turbines had a deposit predominantly found in the form of calcium-aluminum-silicates. This deposit possessed a melting point of 1135 °C. However, in a later study conducted by Borom et al.^24^, the impact of fine environmental particulates on APS-based TBCs was investigated in 1996. Their research revealed that depending on the operating conditions and geographical locations, molten particulates exhibited a threat to the integrity of TBC coatings through spallation. The primary cause of spallation was found to be the incorporation of CaO, MgO, Al_2_O_3_, and SiO_2_ within the molten phase that infiltrates the TBC microstructure, leading to the acronym coined as “CMAS”^24^. However, the discovery of CMAS infiltration posed a significant threat to the GTE, not only for the military aircrafts but also for civil aviation.

Later in 2009, research conducted by Braue highlighted the significant impact of CaSO_4_ (anhydrite) on the structural integrity of high-pressure airfoils, revealing its ability to destabilize yttria-stabilized zirconia at relatively low temperatures^25^. Additionally, their observations of molten CaSO_4_ infiltration without topcoat degradation underscored the potential risks associated with this phenomenon, emphasizing the need for effective mitigation strategies in aerospace applications.

**Fig. 4:**
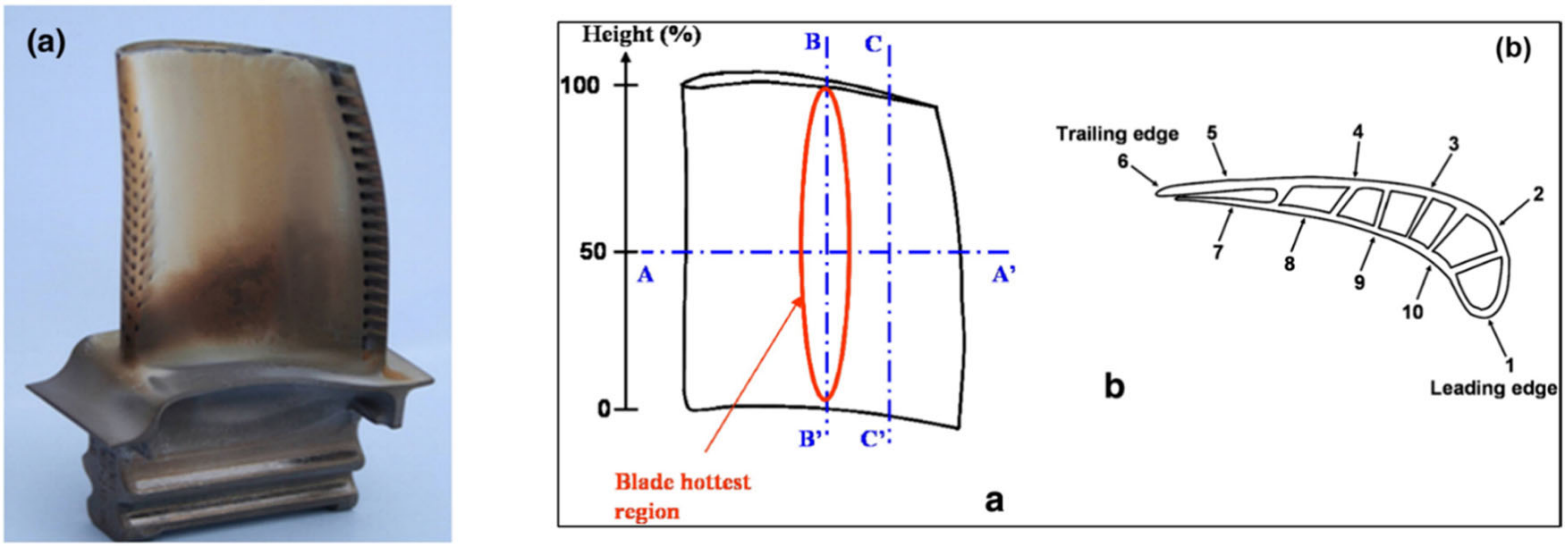
Turbine blades from military engines returned from service show different appearances of CMAS deposits. **a** Photograph of high-pressure turbine blade (pressure side) removed from service, highlighting mix of dark and white deposits and **b** schematics of the pressure side of the blade, indicating the positions of extracted and examined cross sections, as well as the transverse cross-section, highlighting localization markers^26^.

Vidal-Setif et al.^26^ investigated specific sections of the high-temperature and high-pressure turbine blades from military engines that were returned from service, as depicted in Fig. 4a. The researchers noted variations in the thickness, morphology, and infiltration depth of CMAS across different areas of the blade cross-section. The appearance of CMAS differed on the leading edge (labeled as no.1 in Fig. 4b) as well as on the pressure side (labeled as no. 8 and 9 in Fig. 4b). Their study found that the CMAS appears to be porous and inhomogeneous on the leading edge, whilst it was more uniform in the hottest zone of the pressure side. Interestingly, CMAS was not observed on the suction side of the turbine blades (labeled as no. 3 and 4 in Fig. 4b). From this investigation it is clear that the CMAS formation and their infiltration on the turbine blade is greatly dependant on the exposed temperature.

The aerospace industry is confronted with a significant predicament due to the presence of CMAS. Nevertheless, the presence of volcanic ash particles further exacerbates the challenges faced by the industry. For instance, between 1935 and 2003, ~102 aircraft encountered hazardous concentrations of volcanic ash^27^. Level four incidents, meaning “engine failure requiring in-flight restart”, on the severity of encounter, occurred on several occasions. One such incident involved British Airways flight on 24th June 1982, when it flew into an ash cloud from the Galunggung volcano^28^. The engines failed, and the aircraft glided before landing safely in Jakarta, Indonesia. Visibility was severely reduced, and the cabin was filled with smoke and dust^28^. During operation, volcanic ash was ingested into the engine and deposited on the hot sections of the turbine, which result in blocking of the cooling holes and hence, the failure of the components, similar to those of CMAS attack. The volcanic ash is similar in composition to CMAS, but with relatively lower melting temperature, and can cause serious issues for TBC systems^29^.

For over three decades, CMAS has been recognized as a significant menace to aircraft, persisting as a prominent threat even today. Researchers have been working on determining the appropriate chemical composition to use as an analog for the real-world scenario and establishing standardized testing methods in order to better comprehend the real-life effects of CMAS attack/corrosion. Thus far, the studies were extensively focused on their degradation mechanisms on TBCs fabricated by means of APS and EB-PVD technologies. Today, CMAS attacks on TBCs has gained significant attention from both academic and industrial circles. Numerous research centers, universities, and industries worldwide are allocating substantial resources towards this technology. Additionally, there is a growing number of graduate students engaged in CMAS-related studies. Furthermore, gaining a comprehensive understanding of the methods of CMAS formation in aeroengines and how it leads to severe spallation is imperative.

Thus, the scope of this review paper is threefold: firstly, this paper provides a comprehensive review of the chemistry of CMAS and the resulting degradation mechanisms that the TBC system may encounter throughout its service life. Secondly, recent research on the effects of CMAS attack on traditional TBCs is presented and discussed, including the resulting chemical and structural modifications of the topcoat. Lastly, this review examines current strategies and proposes future prospectives aimed at mitigating CMAS infiltration.

## Chemical elements and composition of CMAS

**Fig. 5:**
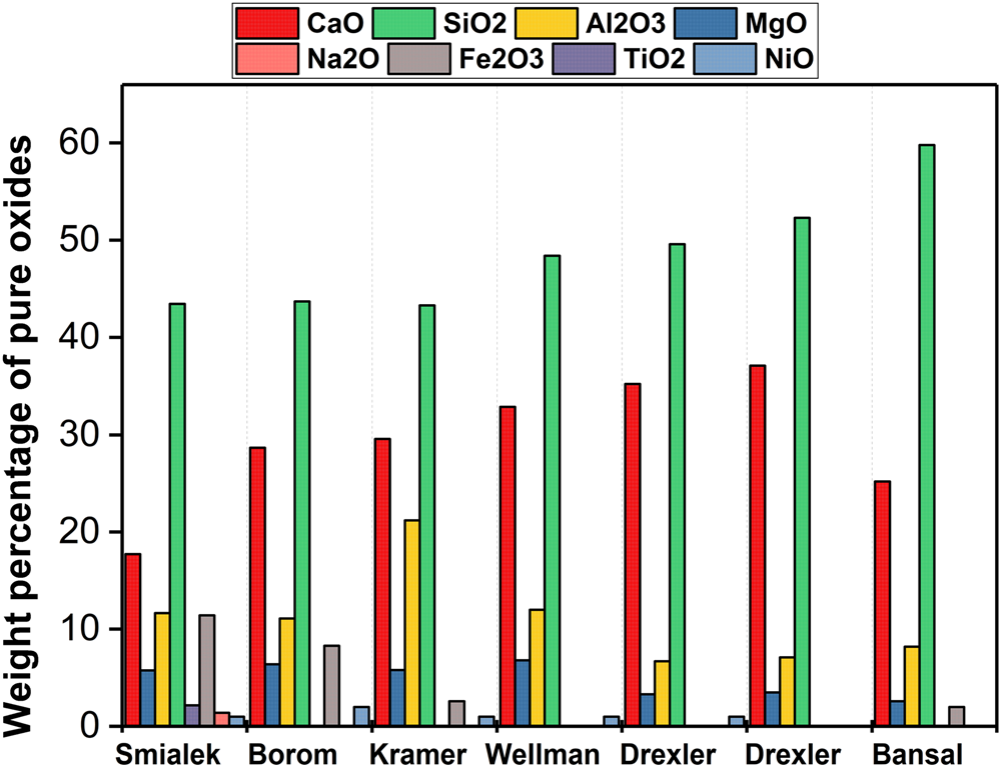
Weight percentage of the chemical composition of CMAS mixture found in different engine components and at different geographic locations, from results published^23,24,32,72,80,109,110^.

CMAS is a type of molten siliceous debris that forms at high engine operating temperatures. It adheres to the surface of ceramic coatings and can cause severe damage to engine components, leading to structural failures. The melting behavior of CMAS varies based on geographical location and the composition of the CMAS itself. For instance, the Air Force Research Laboratory (AFRL) conducted a study that involved collecting sand samples from both the eastern and western regions of the Saudi Arabian Peninsula. Through this investigation, the composition of CMAS was determined, with the predominant presence of mica in the western regions and a higher concentration of dolomite in the eastern regions. This finding confirms that the melting behavior of CMAS varies depending on the geographical location, as mica exhibits a relatively lower melting point (~1300 °C) compared to dolomite (>2300 °C)^30^. Nevertheless, when deposited on actual components, CMAS takes on a glassy, amorphous nature, although some crystalline precipitates have been observed alongside the glassy matrix. These secondary precipitates primarily originate from sand that was not fully melted. Figure 5 exemplifies the chemical composition of CMAS samples that have been studied by the researchers. Smialek et al.^31^ identified the CMAS glass found in the turbine vanes. Soon after, Borom et al.^24^ investigated CMAS glass formed by dirt on the aircraft turboshaft shrouds, which was taken from the different regions of the United States, see Fig. 5. The average CMAS composition observed on aircraft turboshaft shrouds was taken into account by many researchers in order to design a model CMAS chemistry. In 2006, Kramer et al.^32^ showed that CMAS had a chemical composition of 33CaO–9MgO–13AlO_1.5_–44SiO_2_, referred to as C_33_M_9_A_13_S_45_ (expressed as mole percent of single cation oxide formula units). Their study neglected the minor components (i.e., Fe and Ni), believed to primarily emanated from the engine. The model CMAS glass was synthesized by blending precise quantities of reagent-grade oxides, subsequently subjecting them to milling to achieve a thick paste consistency. The thick paste was uniformly coated to the TBC coupons, ensuring a concentration of ~40 mg/cm². The coated coupons were then subjected to a controlled heating process, wherein temperatures ranging from 1200 to 1300 °C, along with a reference temperature of 1400 °C, were maintained for a duration of four hours. The CMAS melted at 1240 °C. Aygun et al.^33^ adapted similar experimental method by using model CMAS glass composition (see Fig. 5) on the APS-based TBC coating. In order to achieve glass formation, the composition was melted at 1500 °C in a box furnace for a duration of four hours, followed by crushing. The process was repeated twice, and the homogenous CMAS precursor was mixed with ethanol and applied to the APS TBC coupons, ensuring 35 mg/cm². The CMAS-TBC interactions were investigated by heat treating the coupons at 1121 °C for 24 hours. The static experiment method was later adapted by many researchers to utilize the model CMAS composition in order to examine the infiltration process and the degradation mechanism of TBCs resulting from CMAS attack.

**Fig. 6:**
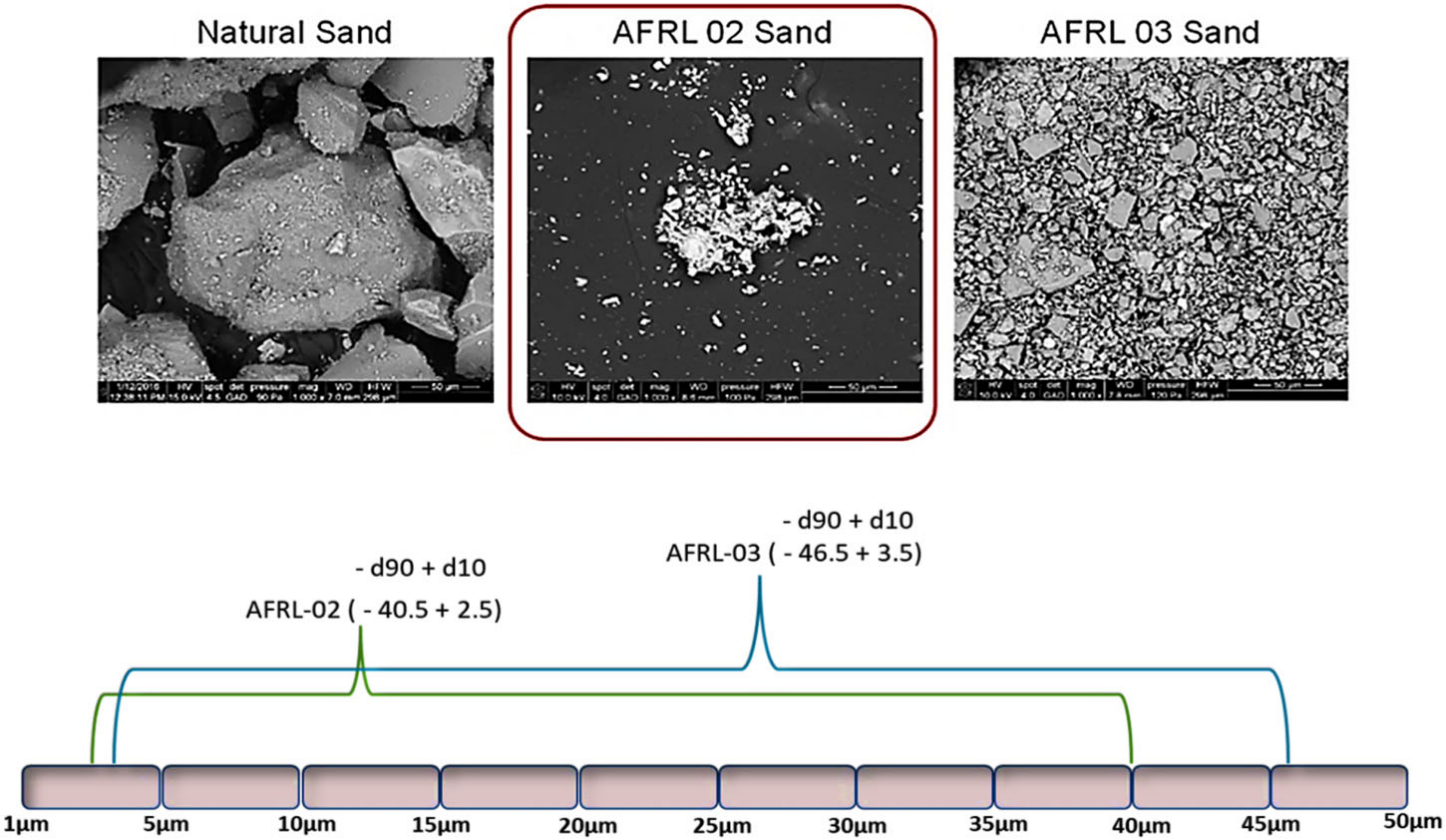
Micrograph analysis of natural sand vs artificial CMAS test media and their particle size distribution. Scanning electron microscope images of natural sand, AFRL-02 sand, and AFRL-03 sand. The morphology of AFRL appears similar to that of natural sand, but different in particle size distribution^34^. The particle size distributions are also shown for both test sands, wherein, the AFRL-03 has a slightly higher distribution.

The US AFRL has developed artificial CMAS test media (i.e., AFRL-02 and AFRL-03) by considering the geological origin, chemical composition, and stoichiometry^34^. The development of these test media involved the application of the standard geo-analytical technique of mineral model analysis to natural engine deposit’s composition. Powder Technology Inc., a trusted commercial supplier based in Burnsville, Minnesota, USA, provides these materials. These test media have been successfully employed in engine dust ingestion tests, showcasing their practical applicability^35^. The morphologies of sand and the developed test media are shown in Fig. 6. The AFRL-02 (bench level) was specifically formulated to produce a deposit resembling CMAS through static laboratory tests. The range of particle size distribution of AFRL-02 is lower than AFRL-03. The AFRL-03 (engine-level) was designed to induce erosion in cold sections and generate CMAS-like deposits in hot sections during full-scale rotating engine tests. The particle size distribution of AFRL-03 is slightly larger in size than that of AFRL-02, making it more suitable for engine-level testing^34,35^.

**Fig. 7:**
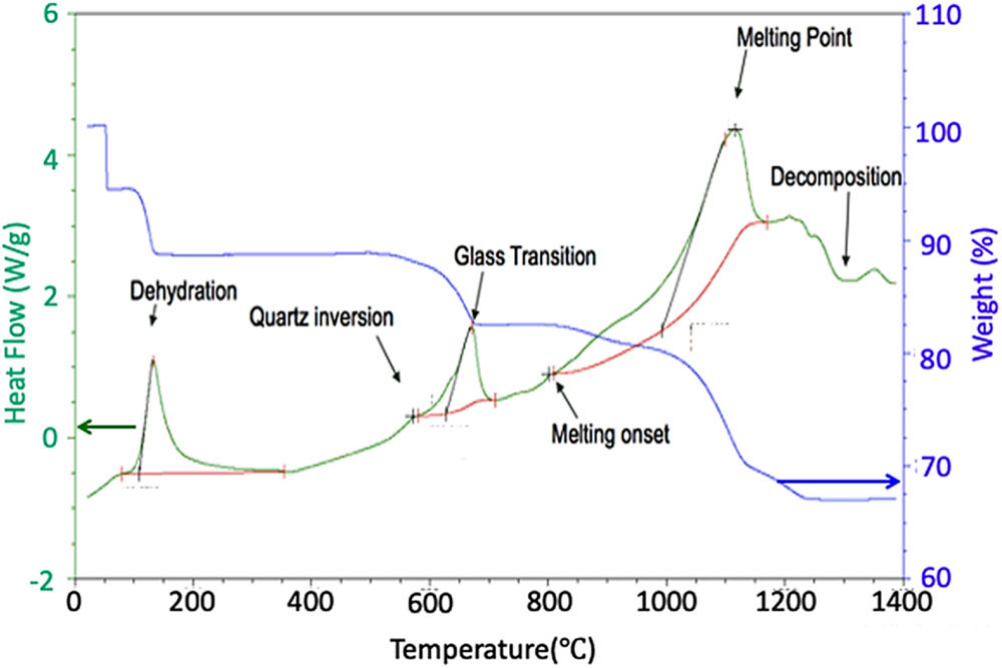
The properties of AFRL-02 CMAS test media using differential scanning calorimetry, revealing thermal transformation. DSC curve of AFRL-02 test sand a peak at 571°C suggesting alpha to beta quartz phase transition and melting initiation at 801°C, followed by CaSO_4_ breakdown at 1116°C^36^.

While the research concentrating on AFRL test CMAS powder is limited, a few researchers have directed their investigations towards the composition of test sand. For instance, examining the AFRL-02 CMAS test powder, Gu et al.^36^ delved into its properties through differential scanning calorimetry, as shown in Fig. 7. This investigation has illuminated a sequence of thermally driven transformations. Within this spectrum, a modest endothermic peak at 571 °C came to light, possibly indicating the shift between alpha and beta quartz phases. The commencement of melting was identified at 801 °C, succeeded by an additional endothermic peak at 1116 °C arising from the breakdown of CaSO_4._ The composition of AFRL-02 powder encompasses a blend of minerals, including 34 wt.% quartz (SiO_2_), 30 wt.% gypsum (CaSO_4_ 2H_2_O), 17 wt.% aplite (NaAlSi_3_O_8_), 14 wt.% dolomite (CaMg(CO_3_)_2_), and 5 wt.% salt (NaCl).

**Fig. 8:**
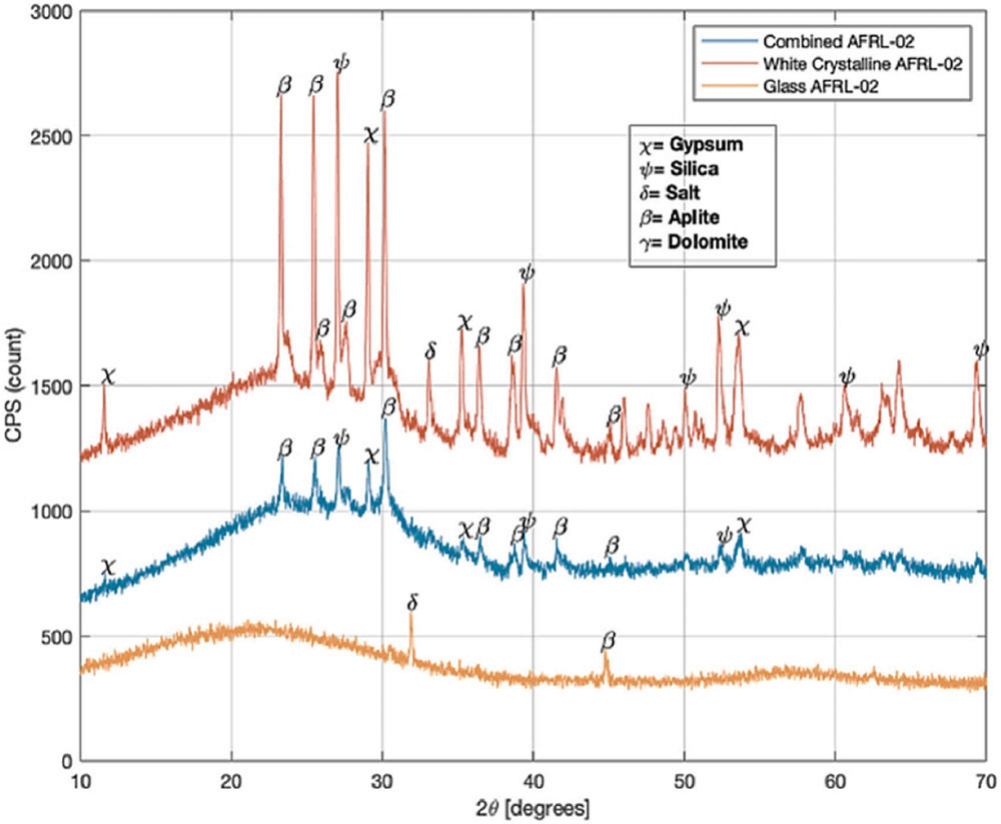
X-ray diffraction spectra for AFRL-02 powder highlighting the crystalline phases including all minerals. The XRD peaks revealed the combination of crystalline and amorphous phases obtained after heating at higher temperature (1500 °C)^37^.

A study by Neito et al.^37^ investigated both the crystalline phases inherent to AFRL-02 and the amorphous glass produced when AFRL-02 is subjected to elevated temperatures. In order to investigate the phases, the researchers heated the AFRL-02 sand at 1500 °C in the furnace. The heating process yielded two distinct outcomes: a white/lavender crystalline phase and a transparent glass, as shown in X-ray diffraction spectra, see Fig. 8. The crystalline material exhibited peaks corresponding to all five minerals present in the initial AFRL-02 mixture. In contrast, the glassy phase demonstrated a wide and diffuse peak indicative of amorphous material, along with smaller crystalline peaks representative of salt and aplite. Through subsequent ball milling, the glassy and crystalline products were combined to create a finely blended frit of glassy and crystalline CMAS formers. The XRD pattern of this mixture confirmed that the dominant component was the amorphous phase, both visually and based on peak area assessments.

## Degradation mechanism of thermally sprayed TBCs

TBCs that function at elevated temperatures (≥1200 °C) commonly experience interaction with molten CMAS, a phenomenon that can result in significant spallation of the TBCs [33]. The phenomenon of delamination and its underlying mechanisms have been extensively investigated by numerous researchers^32,38–40^. The mechanisms of attack appear to be analogous for both the APS and EB-PVD deposited TBCs. Nevertheless, strain tolerance columnar microstructure produced by means of EB-PVD technique induces TYPE 1 cracks at the edges of columns, whilst the voids (i.e., channel cracks and pores) generated during APS method are a governing factor for the corresponding TBC delamination^41,42^. As a consequence, the primary objective of this section is to delve into the degradation mechanism in TBCs produced through thermal spray manufacturing technology, specifically focusing on the impact of CMAS-induced degradation.

**Fig. 9:**
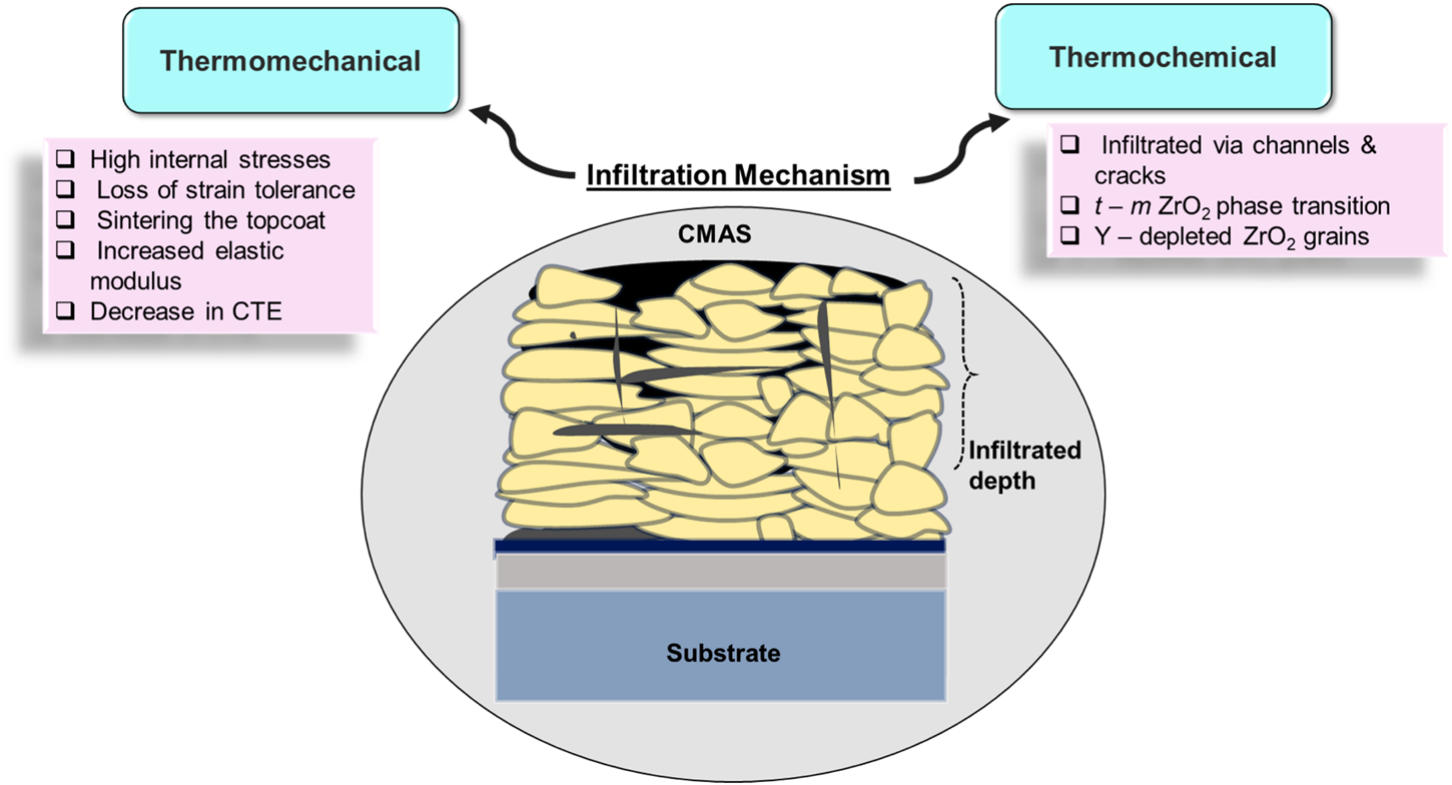
Overview of the degradation mechanisms that impact the spallation of air plasma-sprayed YSZ thermal barrier coatings. Degradation mechanisms for TBCs involves thermomechanical and thermochemical as shown.

At elevated temperatures, molten environmental CMAS not only adheres to the surface of the ceramic topcoat but also infiltrates the microstructure of the topcoat, leading to substantial alterations in its material properties. The degree of deterioration driven by infiltration is governed by a multitude of impactful variables. Yet, a comprehensive discussion regarding the significance of these variables will be presented in conjunction with an exploration of the underlying mechanisms responsible for the degradation process. Degradation mechanisms are generally classified into (i) thermomechanical and (ii) thermochemical mechanisms. A detailed description related to these mechanisms is provided in the next sub-sections. An overview of the factors that impact the failure of APS-based YSZ TBC due to the infiltration of molten CMAS is depicted in Fig. 9.

## Thermomechanical delamination process

**Fig. 10:**
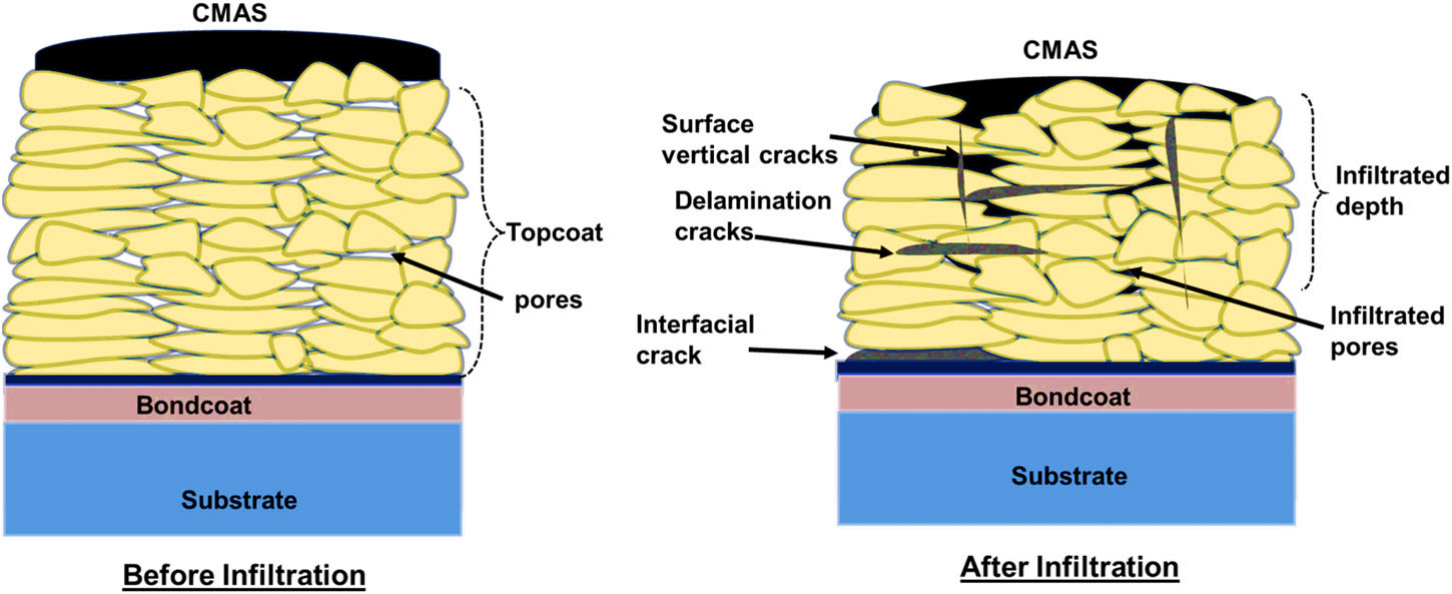
Overview of thermomechanical damage due to CMAS attack on TBC systems. Illustration of thermomechanical CMAS attack on thermal spray TBC coatings, highlighting infiltration of CMAS via cracks, pores and delamination cracks (interlamellar cracks, surface vertical cracks, as well as, interfacial cracks).

The molten CMAS infiltrates directly, leading to a consequential perturbation in the thermal and mechanical properties of the overlying ceramic topcoat. This alteration significantly exacerbates the initiation and propagation of interlamellar, surface, and interfacial cracks, thereby promoting the eventual spallation phenomenon of the TBC structure, as shown in Fig. 10. As CMAS constituents infiltrate and later solidify, significant alterations occur in the ceramic topcoat. These changes include an increase in stiffness, a decrease in strain tolerance, a reduction in the topcoat’s porosity, and an increase in Young’s modulus^42^. This transition in the topcoat ultimately engenders the formation of cracks, leading to the degradation phenomena called spallation.

**Fig. 11:**
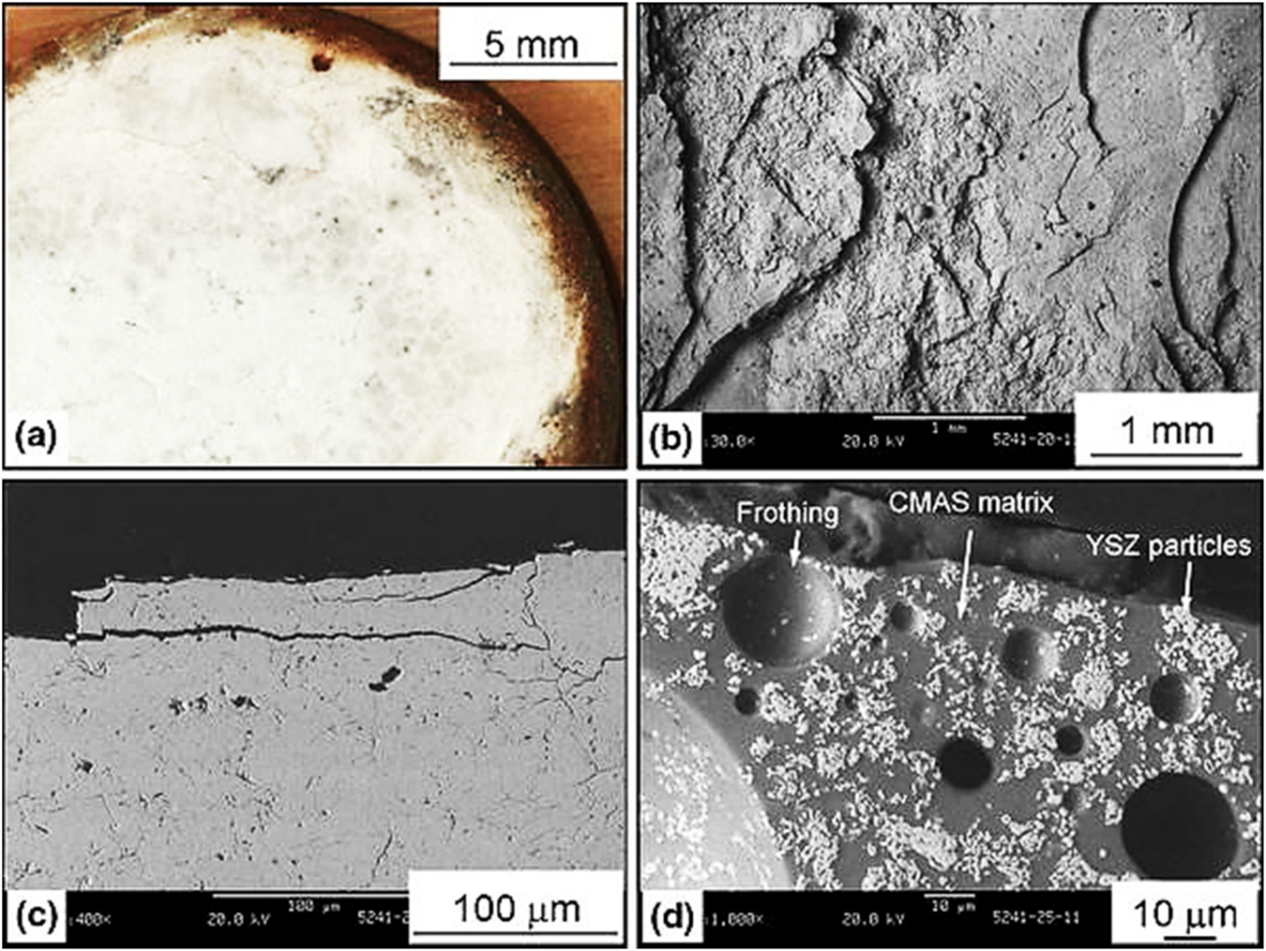
Macro-and-micrograph analysis of TBCs after CMAS attack. **a**, **b** Picture and SEM image of the sample surface showing mud-type flat cracks textures, and **c**, **d** SEM images of the cross-sectional electron micrograph perspectives of the YSZ coating with dense vertical cracks after exposure to CMAS attack. Spallation cracks, and frothing can be seen in cross-sectional views **c**, **d**^44^.

Numerous experimental investigations have demonstrated that the penetration of CMAS does accelerate the process of crack propagation in APS-based ceramic topcoat^38,42–44^. Investigation of CMAS-attacked plasma-sprayed dense vertically cracked YSZ coating revealed that a mud-type flat crack texture was found on the coating surface, as shown in Fig. 11a^44^. Dominant mechanisms underlying the failure of the YSZ coating are spallation cracks, transformation of porous structure to densely composite structure and the occurrence of “frothing”, revealed from the cross-sectional views. These failure modes are shown in Fig. 11. An example of frothing can be seen in Fig. 11d. The emergence of frothing might be attributed to the release of trapped air within the microstructure of the porous coating, which might have trapped within the solidified CMAS layer^42,44^.

**Fig. 12:**
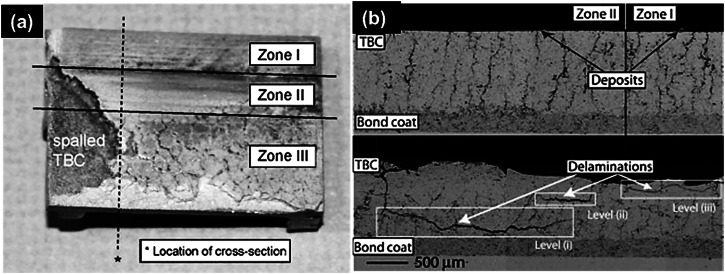
Macro and micrograph analysis of turbine shroud after CMAS exposure. **a** Top view of a segment of a turbine shroud, categorized into different zones (I, II, and III) and **b** according to spallation after CMAS infiltration, and **b** cross-sectional scanning electron micrographs showing delamination zones in TBCs. It is apparent that the three distinct locations of delamination are visible after CMAS infiltration in APS-based YSZ coating^38^.

In the study conducted by Krämer et al.^38^, a detailed observation was made regarding the impact of CMAS penetration on the delamination behavior of TBCs fabricated by means of APS on turbine shroud, as shown in Fig. 12a. The authors categorized the shrouds into zone I, zone II and zone III, wherein, CMAS infiltration and spallation took place in different manner. The investigation revealed the emergence of three distinct locations of delamination: (i) in proximity to the interface of top and bondcoats; (ii) in the vicinity of the CMAS-penetrated layer; and (iii) near the interface between the TC and the deposited CMAS, see Fig. 12b. Severe delamination cracks are visible at the interface between the top and bondcoats, which cause spallation. The occurrence of cracks and delamination are governed by the penetration depth of CMAS into the TBCs, giving rise to localized tensile stress at the surface as it undergoes cooling. The formation of stresses is mainly attributed to the thermal expansion mismatch during cooling. More specifically, owing to the tensile stress, the cracks that originated will propagate through the penetration layer, ultimately leading to the spalling of TBCs. Similar observation was also reported by Cai et al.^45^ for the APS-based YSZ coating, wherein, cracks initiate within both horizontal and vertical CMAS-penetrated microstructures. These cracks seemingly exhibited different geometric sharpness around the regions, where the CMAS layer interacts with the topcoat as well as the bondcoat. As cooling progresses, these cracks propagate, coalescing to induce significant spallation of the TBC from the substrate.

**Fig. 13:**
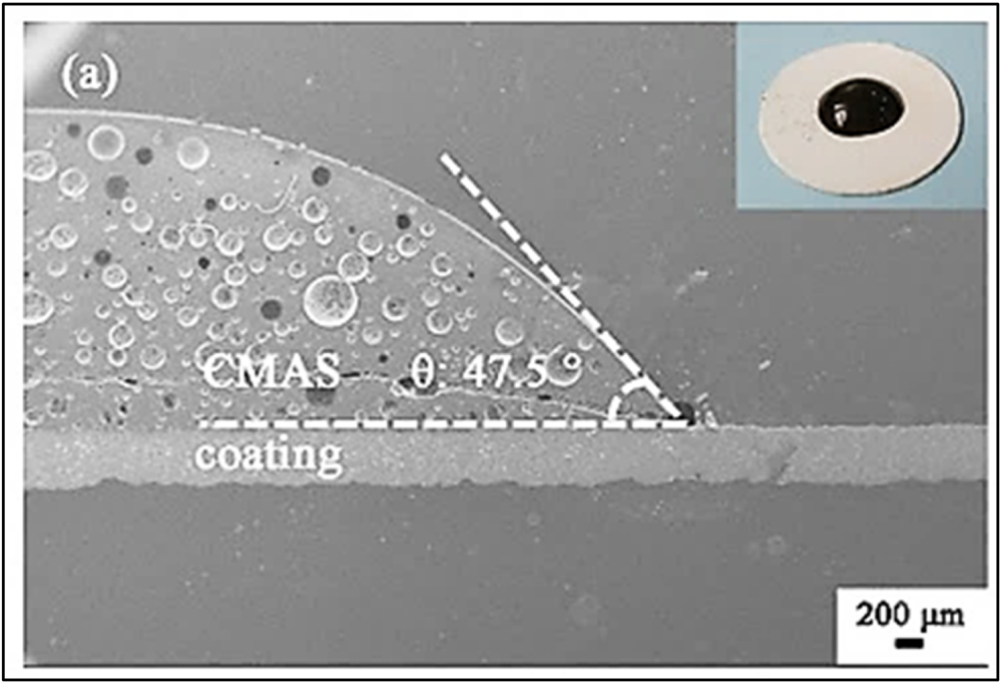
Macroscopic image illustrates the wetting behavior of CMAS and YSZ coating. The CMAS completely melted at 1300 °C after 180 seconds, highlighting contact angle to be approximately 47.50 degrees^47^.

**Fig. 14:**
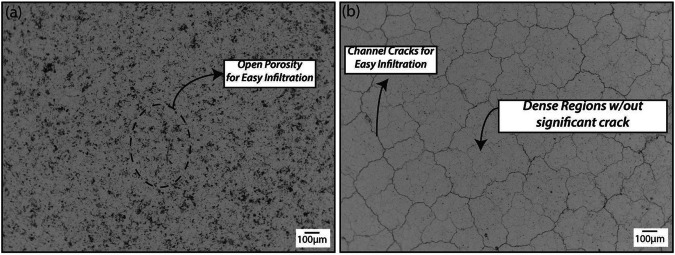
Microscope image illustration of two different morphology fabricated by APS technique. Scanning electron micrographs of the top surfaces displaying **a** porous coating and **b** dense vertically cracked TBC^51^.

The CMAS penetration occurs rapidly on the ceramic YSZ coating surface because of its high wetting behavior^46,47^. For instance, Fang et al.^47^ investigated the wettability of CMAS on the top ceramic coating at a temperature of 1300 °C. The authors reported that the contact angle was measured to be 47.50 degrees, and complete wetting took place after 180 seconds, see Fig. 13. From the inset of Fig. 13, it is understandable that the initial shape of the CMAS melt was partially lost at a temperature of 1300 °C, which highlights the softening and wettability of the CMAS compound. CMAS glass exhibits viscosity of ≤3 Pa.s allowing it to quickly infiltrate the YSZ coating^48^. Due to the exceptional wetting property and low viscosity of CMAS, it rapidly penetrates through open pores, efficiently filling them within the YSZ ceramic coating. Nonetheless, it should be noted that the viscosity of molten CMAS likely depends on temperature gradients and degree of undercooling, which resulted in significant variations through the coating thickness. This can also impact infiltration and consequently delamination^49^. Notably, the YSZ coating produced through the APS process features open pores and channel cracks that facilitate the easy infiltration of molten CMAS. There are significant open channels by means of cracks and porosity, as evident from the micrographs (Fig. 14). These artifacts prevent the accumulation of molten CMAS on the surface of the coatings, allowing it to flow laterally until it finds a clear path to connect these channel cracks. Once the channel cracks have been identified, the molten CMAS penetrates quickly because of capillary action, as reported previously by many researchers^43,48,50–52^.

**Fig. 15:**
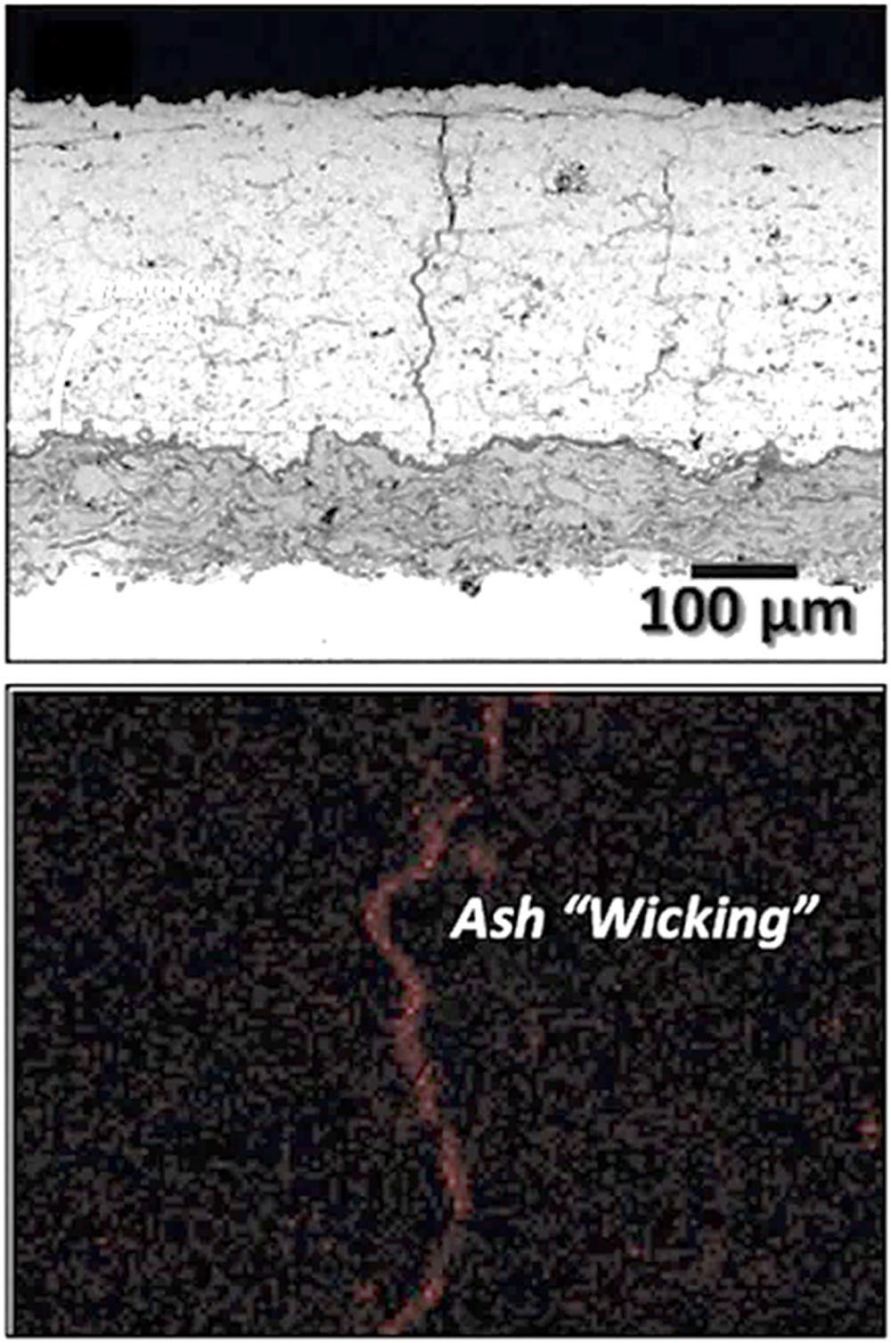
Cross-sectional backscattered scanning electron micrograph of dense vertically cracked YSZ coating produced by means of air plasma spraying. The SEM and EDS revealed that the melted ash infiltrated through the vertical cracks due to the capillary action^51^.

Gildersleeve et al.^51^ examined the penetration of ash melt within the dense vertically cracked YSZ coatings. The authors found that the ash melt follows an infiltration path through vertical cracks of plasma-sprayed YSZ coating, with crack openings ranging from 1 to 2 µm (see Fig. 15). The authors also conclude that the width of the vertical cracks is of the same magnitude as that of EB-PVD coatings, suggesting that capillary forces are likely to favor the infiltration of CMAS melt in both cases. Capillary force or capillary action is a phenomenon of liquid movement within the spaces of a porous material owing to the forces of adhesion, cohesion, and surface tension. The molten CMAS penetration within the ceramic topcoat is likely governed by the capillary action, as reported previously by various researchers. Furthermore, a higher concentration of CMAS melt within the topcoat potentially leads to elevated stress concentrations, resulting in the significant delamination of the coating. This infiltration can lead to the expansion of APS-coated TBC in the vertical direction. Therefore, the infiltration depth with respect to time serves as the sole dependent factor to consider in the CMAS attack mechanism. According to Shan et al.^53^ when a CMAS-attacked TBC shows a measured infiltration depth of ~100 μm, it does not necessarily imply that the entire top 100 μm of the original APS-based TBC has been infiltrated. To estimate the thickness of the infiltrated as-sprayed TBC, one should deduct the thickness of the unaffected zone from the initial TBC thickness (~250 μm). When CMAS partially penetrates the TBC, the topcoat can be conceptualized as a two-layer TBC (one with a penetrated layer and another one with a non-penetrated layer). In this arrangement, the CMAS-infiltrated layer exhibits a relatively higher Young’s modulus compared to the non-infiltrated layer^43^. It has been reported that Young’s modulus with the CMAS-penetrated layer YSZ coating exhibits ~250 GPa compared to the non-penetrated 8YSZ coating (40 GPa)^47^. Additionally, due to CMAS penetration, the apparent porosity appears to have significantly decreased (from 25% to 5%) in the plasma-sprayed YSZ topcoat, leading to an increase in thermal diffusivity^54^. As a consequence, the top ceramic coating is stiffened accompanied by a reduction in strain tolerance as well as loss of CTE, and densification, ultimately leading to crack propagation and spallation.

Based on this multitude of variables such as pore concentrations, capillary forces, CMAS viscosity, and surface tension, the time taken for the degree of infiltration depth has been determined by many researchers^54^. One such example is by incorporating these variables in an equation given below^32^.

1 $$t=\left[\frac{{k}_{t}}{8{D}_{c}}\,{\left(\frac{1-w}{w}\right)}^{2}{L}^{2}\,\right]\frac{\eta }{{\sigma }_{{LV}}\,}$$

where *η* is the CMAS glass viscosity, ${\sigma }_{{LV}}$ is the glass surface tension, *k*_t_ is the tortuosity of TBC pores, *w* is the porosity open to flow, and *D*_c_ is the capillary diameter. Based on the equation, as reported by Wu et al.^54^, the infiltration time (*t*) was measured to be 4 hours, resulting in an infiltration depth of 400 µm. In contrast, the CMAS penetration time takes 22 hours to complete a 1000 µm thickness for the YSZ coating. It is to be noted that as the temperatures drops at the TBC surface, the reduced viscosity of the molten CMAS resulted in lower rated of infiltration, which is governed by the thermal gradient and degree of undercooling. These variables, therefore, help researchers focus on arresting the CMAS infiltration.

## Thermochemical delamination process

**Fig. 16:**
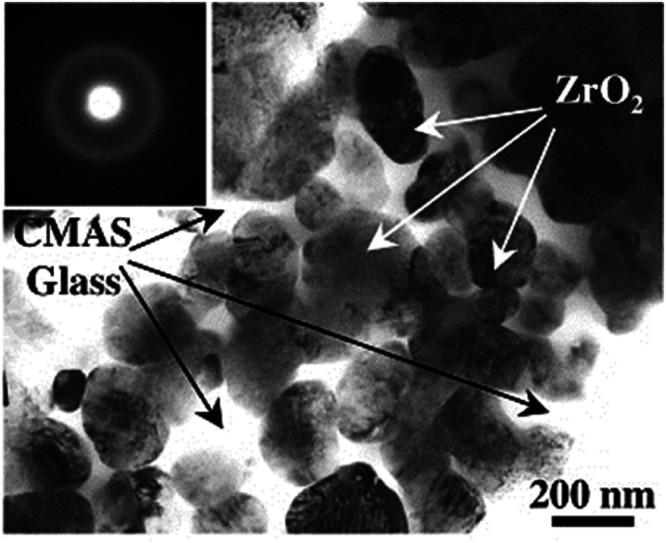
Micrographic image illustration showing the morphology of TBC after CMAS exposure. Transmission electron micrographs highlighting the globular ZrO_2_ grains formation within the CMAS glass^33^.

Although delamination cracks and spallation remain as the primary failure modes of YSZ coating resulting from CMAS, substantial structural deterioration of the YSZ coating can also emerge due to phase transitions resulting from interactions between CMAS and TBCs. Krämer et al.^32,38^ conducted a study on the thermochemical deterioration of YSZ coatings, revealing that the interaction between zirconia and CMAS compounds can lead to a phenomenon called “re-precipitation” or “dissolution-precipitation mechanism”. This interaction triggers the formation of yttria-depleted ZrO_2_ grains. Due to this re-precipitation process, the stabilizing influence of yttria on zirconia is diminished or nullified. Consequently, zirconia undergoes an allotropic transformation, signifying a change in its crystal structure. This transformation arises because zirconia is no longer being stabilized by yttria. The occurrence of reprecipitated yttria-depleted ZrO_2_ grains takes place in the form of globular grains, embedded in the CMAS glass^33^, as shown in Fig. 16. The nature of such globular re-precipitation ZrO_2_ grains has been reported by many researchers, and it greatly depends on the local melt chemistry of CMAS^32,33,43^.

**Fig. 17:**
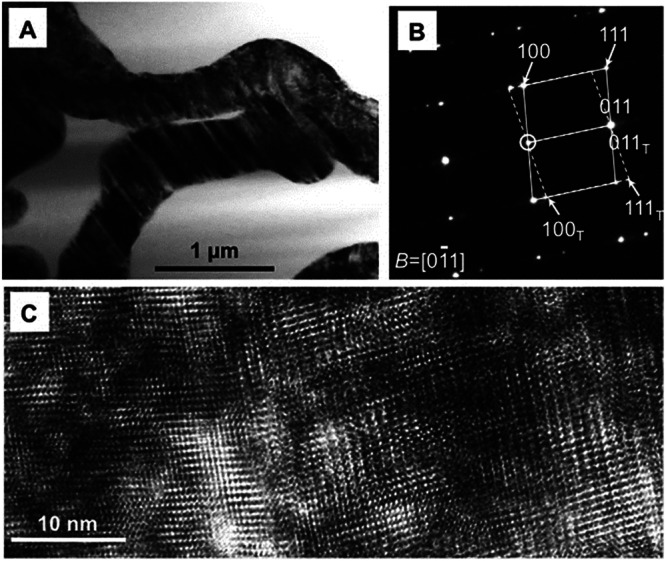
Micrographic image illustration showing the morphology of TBC after CMAS exposure. **a** High magnification transmission electron microscopy (TEM) image reveals the grains on the surface of the 7YSZ sample after it was exposed to CMAS at 1340 °C for 24 hours; **b** selected area electron diffraction pattern (SAED) of the *m*-ZrO_2_ grain within the image in **a**. In this pattern, ‘B’ designates the zone axis, and the transmitted beam is encircled. Inside the dashed box, you can observe corresponding reflections marked with the subscript ‘T,’ which indicate mirror-like reflections resulting from twinning; **c** a high-resolution TEM image displaying a twin boundary originating from one of the grains (**a**)^43^.

For instance, Krause et al.^43^ investigated the high-temperature interactions between CMAS glass and an APS-based 7YSZ coating by subjecting them to temperatures of 1340 °C for both 24 and 72 hours, respectively. The investigation revealed that the phase did not undergo complete transformation during the interactions. Instead, after 24 hours of heat treatment, the top 45 µm exhibited a transition from the *t*-ZrO_2_ tetragonal phase to the *m*-ZrO_2_ monoclinic phase. Conversely, after 72 hours of CMAS interactions, significant traces of the *m*-ZrO_2_ monoclinic phase were found within the topcoat. Figure 17 confirms the phase transition from *t-*ZrO_2_ to *m*-ZrO_2_, which revealed that the ZrO_2_ grains underwent significant twinning after CMAS interactions. More specifically, *t-*ZrO_2_ grains undergo dissociation by introducing Zr^4+^ and Y^3+^ to the melt CMAS at a temperature beyond 1300 °C. As a result, the Zr^4+^ ions move quickly, so they reach the maximum amount that can be dissolved in the CMAS glass rapidly. This enables re-precipitation with the emergence of yttria lean ZrO_2_ grains. As the exposure time increases to 72 hours, the CMAS fully penetrates the topcoat, leading to the diffusion of Y^3+^ ions into the extensive CMAS glass volume. This, in turn, results in reducing the yttria content locally, thereby transforms into the yttria-depleted *m*-ZrO_2_ grains upon cooling^43^. Zhou et al.^55^ also claimed that the CMAS typically attacks the YSZ grain boundaries, resulting in a dissociation precipitation mechanism. Because of the higher diffusion coefficient of yttria than that of Zr, depletion of yttria took place, which leads to the occurrence of yttria lean Zr grains. This results in diffusion of Ca^2+^ and Mg^2+^ ions into Zr, which neglect the stabilization of Zr, leading to monoclinic phase transition. Similarly, Mohan et al.^56^ observed the occurrence of destructive yttria-depleted *m*-ZrO_2_ grains during the cooling process. Nevertheless, the authors argue that the phase transitions from *t-*ZrO_2_ to *m*-ZrO_2_ predominantly took place on the top surface of the YSZ coating rather than the lower surface. This phenomenon can be ascribed to the increased concentration of yttria content in the melt CMAS, which enables the dissolution-precipitation of Y_2_O_3_-rich cubic ZrO_2_ phase in close proximity to the lower portion of the YSZ coating.

**Fig. 18:**
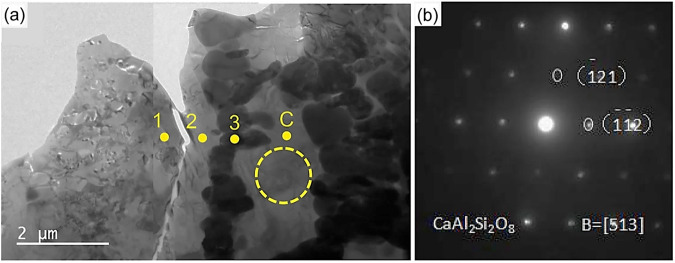
Micrographs of interface Analysis between top-and-bond coats, confirming anorthite product. **a** Bright-field micrograph taken from the interface region between ceramic layer and bond coat. The point 1 and 2 represents the CMAS infiltration, while point 3 represents the YSZ coating. C represents the composition contains anorthite product (26.07CaO-37.62AlO_1.5_-36.31SiO_2_ (mol.%)); and **b** SAED pattern indicating the anorthite phase taken from point C^57^.

Another study conducted by Wu et al.^57^ examined thermochemical reactions between the CMAS and APS-based YSZ coating by heating at 1250 °C for 8 hours. The melting point of CMAS was 1178 °C. The investigation unveiled the presence of two novel reaction products. A Zr_2_Y_2_O_7_ phase materialized due to the localized arrangement of Y^+^ ions within the YSZ coating, contributing to the preservation of the *t*-ZrO_2_ phase. However, within the midsection of the YSZ coating, no evidence of the stabilized phase was identified. Instead, a fresh reaction product known as anorthite (CaAl_2_Si_2_O_8_) emerged, characterized by increased levels of calcium and aluminum (see Fig. 18). This anorthite product displayed a melting point of 1553 °C and possess a solid nature of crystalline precipitation^33^. The appearance of anorthite detrimentally impacted the performance of the YSZ coating as it filled the interlamellar gaps, diminishing strain tolerance and ultimately leading to the formation of microcracks. Interestingly, the authors did not observe any destructive *m*-ZrO_2_ monoclinic phase as evidently seen by other researchers. As discussed above, the reaction products or phase transition is likely depending on the local melt chemistry of CMAS. Nevertheless, there are reports suggest that anorthite crystalline product serve as a mitigation process arresting the melt CMAS infiltration^58^. The role of how the anorthite crystalline phase arresting the melt CMAS is still under debate.

The anorthite (CaAl_2_Si_2_O_8_) phase occurs at temperature ranges between 1000 and 1240 °C by self-crystallizations along with diopside (CaMgSi_2_O_6_) and wollastonite (CaSiO_3_), as reported previously by Zhang et al.^59^. The authors claimed the anorthite formation requires extended incubation period characterized by a lower temperature threshold in comparison to the other two resultant compounds. Furthermore, the emergence of anorthite is influenced by the higher Al content, in conjunction with Ca and Si concentrations. In contrast, the preferential formation of diopside is facilitated by the presence of magnesium in the product due to its increased affinity for calcium. Another possible rationale may stem from the presence of substantial fractions of diopside, which seemingly appears to favor the formation of anorthite, leading to the accumulation of aluminum in the vicinity of diopside.

## Pore characteristics on the CMAS infiltration

Atmospheric plasma-sprayed TBCs exhibit microstructural artifacts such as pores, cracks, and splat interfaces. It has been reported that the pores, cracks, and splat interfaces are aligned perpendicularly to the heat flux direction, contributing to the thermal insulation performance of the TBCs^60^. The overall porosity of APS-based 8YSZ coating typically ranges between 15% and 25%^61^. Nevertheless, the infiltration of CMAS occurs through these pores and channel cracks by reducing the overall porosity and, ultimately, declining the thermal insulation performance. Reports suggest that some pores remain un-attacked by the melt CMAS. Therefore, this section provides a comprehensive review of the interactions between the CMAS melt and pore characteristics.

A study conducted by Shan et al.^62^ examined the pore features of YSZ coating fabricated by means of APS. CMAS paste was applied on the TBCs, and heat treated at 1250 °C for three distinct durations (i.e., 0.5, 1, and 3 hours). The APS YSZ coating exhibited different microstructural artifacts such as intersplat and intra-splat cracks, and globular pores, with a total apparent porosity of ~11.4%. CMAS melt was not completely infiltrated within the topcoats after 0.5 and 1 hours, whereas a complete penetration of CMAS melt was found after 3 hours. The authors reported that the channel cracks and network pores, as well as a few globular pores were filled with CMAS. Indeed, the crack networks disappeared by densifying the topcoat, with a decrease in overall porosity from 11.4% to 4.8%. The plausible explanation for this phenomenon could be due to the closed globular pores, wherein, the CMAS was not able infiltrate within the span of 3 hours by attacking grain boundaries of YSZ. Notably, an increase in porosity was found in the region corresponds to top regions, where the CMAS attacked severely. The authors claim that the increase in porosity in the section is mainly attributed to the splat separation. An increase in splat gaps up to 3.9 µm was found for CMAS-infiltrated YSZ coating. These gaps could result from tensile stresses generated from thermomechanical and thermochemical coupling mechanisms^62^.

Perhaps influenced by the presence of large unfilled pores, the same group investigated the CMAS infiltration of APS-based YSZ coatings by tailoring it to produce large pores and porosities as high as 23%^53^. The CMAS test was conducted with similar parameters as mentioned previously. The authors noted that the CMAS infiltration and separation of splats were sluggish for the coatings with large pores compared to the YSZ coatings with total porosity <10% after 3 hours.

**Fig. 19:**
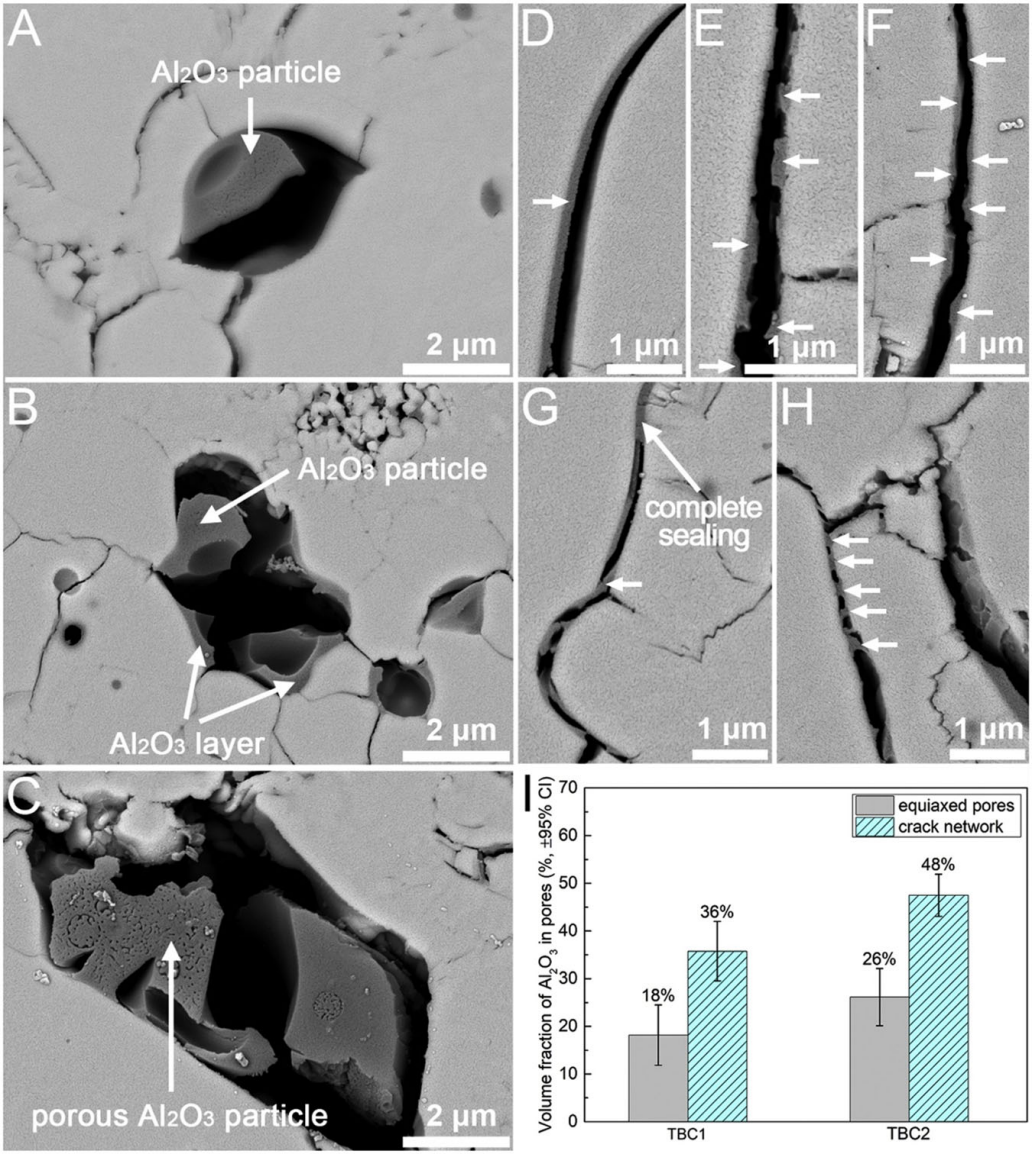
Micrographic analysis of Al_2_O_3_ particles embedded within cracks and pores of thermal barrier coatings. Backscattered cross-sectional scanning electron micrographs showing Al_2_O_3_ particles embedded inside the crack network pores and equiaxed pores, noting **a**, **b** dense Al_2_O_3_ particles, **c** a porous Al_2_O_3_ particle inside the pore and **d**–**h** high-magnified images showing the particles embedded within the microcracks, providing complete shielding^63^.

**Fig. 20:**
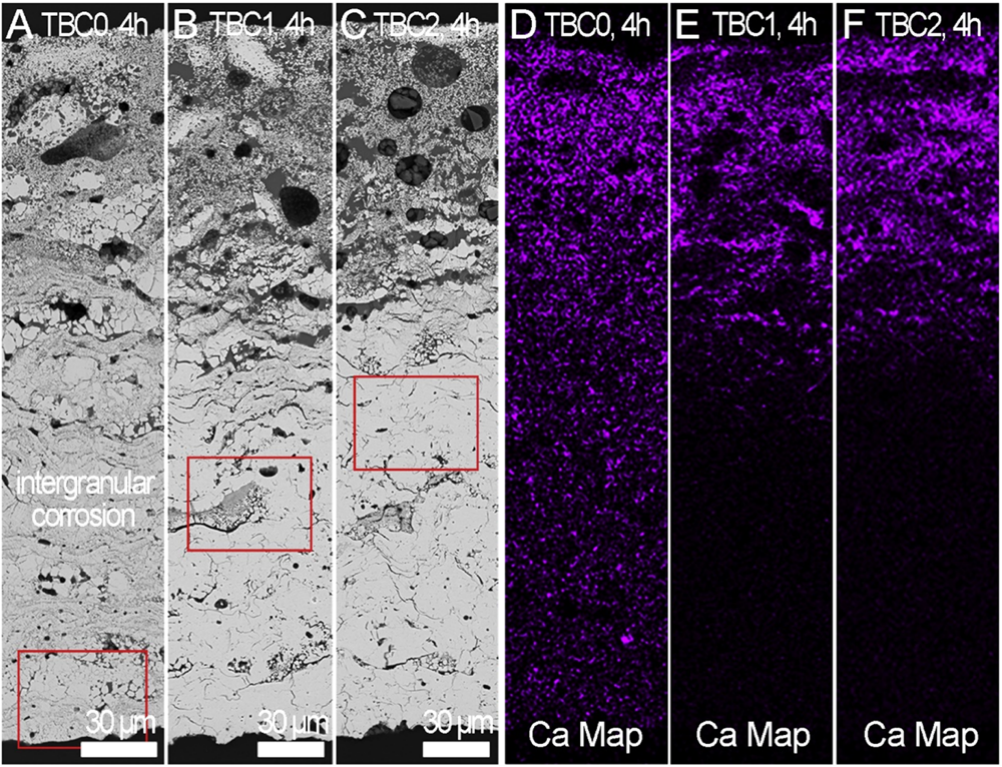
Micrographic analysis on how Al_2_O_3_ particle impregnation mitigate the CMAS infiltration. Backscattered cross-sectional scanning electron micrographs showing **a** TBC without Al_2_O_3_ particle impregnation, **b** TBC with one impregnation run, and **c** TBC with two impregnation runs. Corresponding EDS maps can be seen in the figures **d**–**f** indicating that TBC0 is completely infiltrated with CMAS, causing intergranular corrosion, whereas the other two Al_2_O_3_ particle impregnation TBC coatings (TBC1 and TBC2) exhibited about half the thickness of CMAS infiltration^63^.

Generally, the pores in the YSZ coatings are interconnected, and form a network called network pores. Owing to these network pores, CMAS infiltrates rapidly, causing topcoat delamination. In order to mitigate the CMAS infiltration, Shan et al.^63^ explored the pore characteristics of YSZ coating through tailoring with Al_2_O_3_-sol impregnation. The impregnation was carried out in a vacuum chamber with low pressure (0.01 MPa). The authors chose Al_2_O_3_ because of its capability of arresting CMAS melt infiltration. The CMAS test were performed for 4 hours. The Al_2_O_3_ particles were clearly visible in the regions of equiaxed and crack network pores, wherein a few particles were observed as a dense structure, whilst some porous Al_2_O_3_ particles also can be seen in the equiaxed and network pores, see Fig. 19a, b. CMAS studied revealed the evidence of intergranular corrosion, indicating a complete penetration of YSZ coatings without any Al_2_O_3_ impregnation (Fig. 20a). There is no evidence of complete penetration of CMAS was found for the YSZ coatings with Al_2_O_3_ impregnation after 4 hours, see Fig. 20b, c. This suggests that the pore-modified Al_2_O_3_-sol impregnation helps reducing the CMAS infiltration throughout the YSZ coating. Al_2_O_3_ within the network of cracks and equiaxed pores reacts with CMAS melt, resulting in the formation of elongated anorthite reaction products within the network pores. These anorthite products bridge the gap between the crack network pores, effectively reducing the likelihood of further infiltration.

## Significance of optical basicity study

The optical basicity (OB) (denoted by Λ) was first introduced by Duffy^64^ in order to categorize the chemical reactivity of oxides in glass. The theoretical background of OB relies on orbital expansion effects that reveal the chemical bonding nature between a Lewis acid-base pair. Generally, the oxygen behaves as Lewis’s base, whilst the metal ions as Lewis’s acids. The concept of OB pertains to the reactivity of oxygen atoms within the glass structure, particularly in response to the acidic influence exerted by solute metal ions. More specifically, it is determined by the ability of oxygen anions to donate electrons, and this capacity is influenced by the characteristics of the metal cations. Indeed, cations with low polarizability (i.e., high OB) enable oxygen to readily donate electrons to the available cations. Oxygen ions are highly effective at donating electrons when they exist independently. However, when they form bonds with other ions, like silicon (Si^4+^), their electron-donating ability diminishes. This effect is more pronounced when oxygen ions bond with highly polarizing cations, such as Si^4+^. These cations draw electrons away from the oxygen ions, making it difficult for the resulting compound (e.g., SiO_2_) to donate electrons elsewhere. As a result, in the context of materials like CMAS, CaO and MgO are considered basic oxides because they readily donate electrons, while silicon dioxide (SiO_2_) is an acidic oxide. Al_2_O_3_ exhibits amphoteric behavior, meaning it acts as a base in the absence of other bases but behaves as an acid in the presence of basic oxides such as CaO or MgO^22^. The OB values of CaO, MgO, Al2O3, and SiO2 are 1.00, 0.78, 0.60, and 0.48, respectively^65^.

**Fig. 21:**
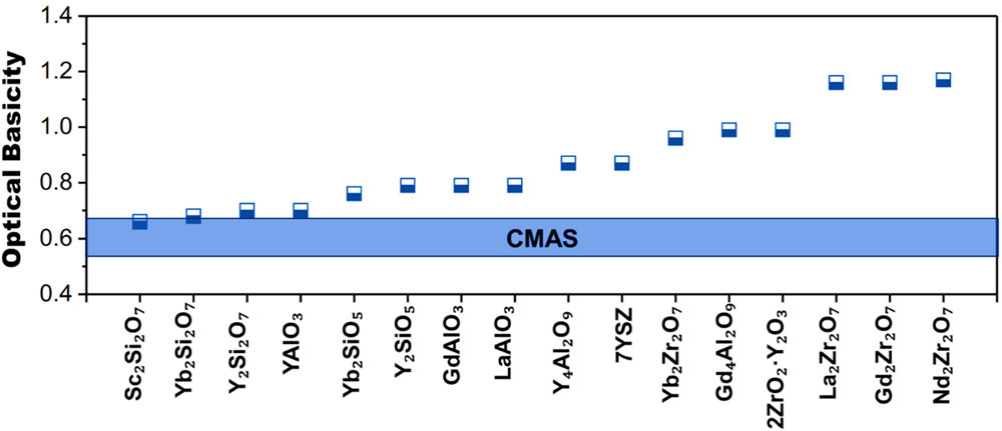
Optical basicity (Λ) of various thermal barrier and environmental barrier ceramic materials. The blue bar represents the range of CMAS, which is typically between 0.49 and 0.65^111^.

The notion of OB has been widely used by researchers in order to better comprehend the significance of CMAS attack on TBCs. The OB value (Λ) can be theoretically calculated using following equation

2 $$\Lambda =\sum i{X}_{i}{\Lambda }_{i}$$

where ${X}_{i}$ and ${\Lambda }_{i}$ are the mole fraction of the *i*th constituent of the compound and its OB. According to the theory of OB, a significant difference in the value of OBs between CMAS and the ceramics highlights a severe corrosion attack^66–68^. Figure 21 shows the OB of well-known thermal barrier ceramic materials, in conjunction with CMAS formers. Typically, CMAS exhibits an OB ranging between 0.49 and 0.65, highlighting that the CMAS behaves as a basic oxide^29^. The YSZ ceramic coatings exhibit the OB value of around 0.85, with a threshold limit of 20% with CMAS. On the other hand, environmental barrier coatings (EBCs) typically ranges between 0.6 to 0.8. The OBs of Gd_2_Zr_2_O_7_ and Nd_2_Zr_2_O_7,_ the most reactive of all rare earth zirconates with value of 1.2^29^. The greater the difference in OB with CMAS, the greater the reactivity. Kumar et al.^69^ demonstrated that yttrium aluminum garnet (YAG) TBC coating exhibit OB values of around 0.70 as compared to that of traditional YSZ coating. Hence, the difference between the YAG and CMAS is lower, which in turn help reducing the reactivity and associate damage for the YAG TBC material.

A recent study by Turcer et al.^66^ delved into the examination of EBCs for their resistance against CMAS attack. YAlO_3_ and Y_2_Si_2_O_7_ were selected as primary choice of materials for their investigation. The authors established a screening criterion to select CMAS-resistant EBC, focusing on their OB values. The OB difference between CMAS and the chosen EBC material was minimal, as shown in Fig. 21. The study findings revealed that yttrium could induce extensive reaction-crystallization without CMAS glass penetrating the grain boundaries. Krause et al.^29,50^ conducted studies that corroborated the precipitation of high yttria compounds upon interaction with molten CMAS for the 2ZrO_2_.Y_2_O_3_ TBCs. Their findings align closely with OB, underscoring the consistency between observed precipitation behavior and OB in this context.

A study by Li et al.^70^ investigated CMAS corrosion attack on A_6_B_2_O_17_ (where A = Zr, Hf; B = Nb, Ta) for TBCs. The microstructural implications after the melt CMAS attack were correlated with the OB study. The study revealed that the Ta and Nb mainly reflected greater reactivity with CMAS melt, with dissolution/re-precipitation of matrix into the residual CMAS, highlighting the occurrence of dense layer and reduced degree of porosity. They reported that the microstructure obtained after CMAS attack greatly depends on the theoretical OB value. More specifically, the Ta_2_O_5_ and Nb_2_O_5_ displays a higher OB value of 0.94 and 1.05, respectively. This suggests that a greater difference in the OB values between the CMAS and tested TBCs, can lead to reverse dissolution. Indeed, owing to higher OB value of Nb, the reactivity is more intense, which leads to the formation of a dense layer after CMAS attack. OB plays a pivotal role in predicting the reactivity between TBCs and molten CMAS. Contemporary researchers strategically modify the TBC composition, and even EBCs guided by OB theory, aiming to mitigate reactivity. Despite these advancements, infiltration through grain boundaries poses a risk of inducing cracking. This theoretical approach signifies the forefront of innovative thinking in integrating EBCs with TBCs, emphasizing resistance to CMAS infiltration.

## Dynamic interactions of CMAS droplets

The predominant focus in the extensive research conducted on the interaction between molten CMAS and TBCs has centered on static conditions, specifically through furnace exposure. Although the examination under static conditions has contributed significantly to the fundamental aspect of the microstructural consequences of CMAS attack, there remains a gap in understanding the crucial behavior of TBC microstructures when subjected to dynamic conditions that replicate real engine-relevant scenarios. The envisioned GTE setting is far from straightforward, characterized by high-speed non-steady state combustion flows. By employing dynamic interaction rigs offer a significantly more authentic environment by replicating not only the precise chemistry or temperature ranges but also approaching the integrated thermomechanical conditions encountered. Despite this, research on dynamic interactions is notably lacking, creating an opportunity for researchers to delve into the microstructural implications using diverse experimental methods.

**Fig. 22:**
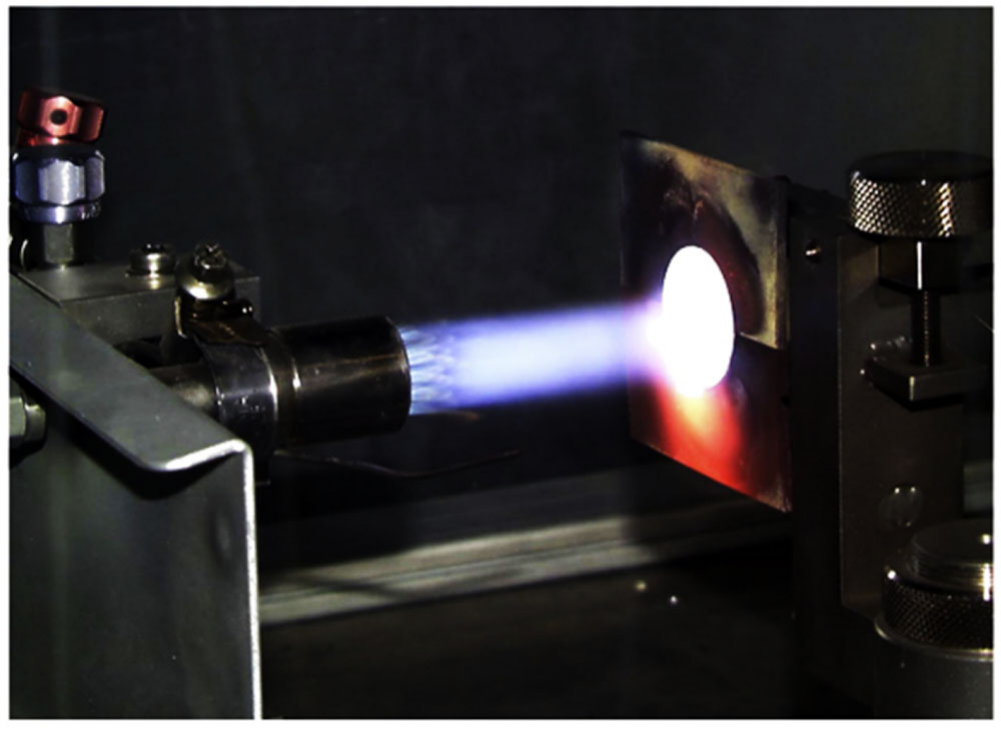
Photograph of burner test rig for CMAS frit testing on a TBC coupon sample. In the burner test rig the CMAS test media prepared as suspension can be sprayed axially to the center of the flame once the temperature set of 1200 °C ^71^.

Dynamic interactions are typically carried out using burner test rigs by researchers. For instance, Steinke et al.^71^ utilized an intriguing approach to test CMAS frit on the TBC with the help of thermal gradient cycling testing, as depicted in Fig. 22. The burner was operated with a natural gas/oxygen mixture to produce a light blue flame. CMAS was prepared as suspension by mixing the CMAS frit in deionized water. The CMAS suspension was fed axially to the center of the flame once the TBC reached the temperature of ~1200 °C. In this study, the authors investigated multilayered YSZ/Mg spinel TBC fabricated by means of APS. The study reported that the direct injection of the CMAS solution through the central axis of the burner nozzle performed well in terms of achieving a uniform and particularly continuous CMAS loading of the test specimens during each test cycle. The CMAS penetration was only near-surface at a depth of ~20–30 μm from the coating surface. Drexler et al.^72^ also conducted similar research, utilizing a burner test rig to dynamically interact CMAS droplets with YSZ+Al+Ti coatings fabricated through solution-precursor plasma spraying. Surface temperature monitoring via a pyrometer was employed. Once the surface temperature reached 1235 °C, a mixture of CMAS powder and deionized water was sprayed axially into the natural gas/oxygen flame. The heating cycle was maintained for 5 minutes, followed by a 2-minute air cooling cycle. Their findings indicated that the mitigating mechanisms observed were similar to those reported in furnace isothermal testing.

**Fig. 23:**
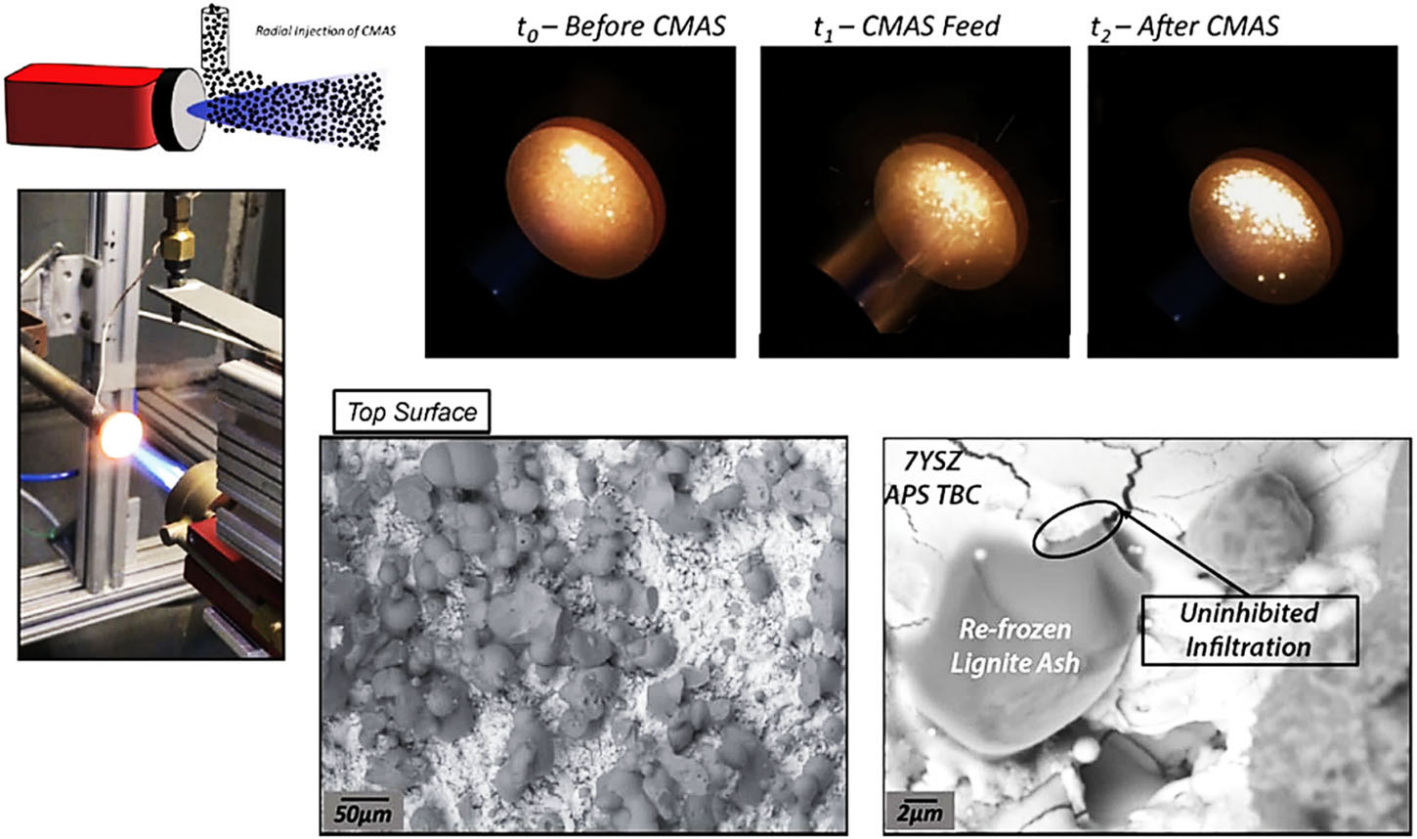
Experimental overview of gradient test rig, wherein, the CMAS has been injected into the flame radially. In this regard, the specimen can be tested for dynamic interactions of CMAS splats and the TBC surface. The SEM images revealed the top surface after CMAS feeding, and corresponding higher magnified SEM shows the interaction between the CMAS and YSZ coating^52^.

A team from Stony Brook University in the USA also investigated how the coating surface responds to impacting CMAS droplets^51,52^. They provided a brief explanation of their custom burner test rig to enhance understanding. The use of burner rig tests provides a mechanism to better simulate the actual operating conditions. Figure 23, the gradient test rig setup is depicted, controlled by mass flow controllers and a LabVIEW application. The temperatures of TBC sample surfaces were measured using a thermopile pyrometer, and the corresponding backside temperature was monitored with a type-K thermocouple. The experiments used a burner torch with both small and large combustion flames. However, a smaller flame was selected to better mimic the localized dynamic and gradient conditions influenced by the intricate geometries and dimensions of actual turbine components in the engine. The torch was positioned at a stand-off distance of approximately 60 mm from the sample surface. The samples were preheated to achieve an average surface temperature of 1275 °C before introducing the CMAS. Once the desired temperature was reached, CMAS powder was applied at a low loading capacity of about 10 mg/cm^2^. After depositing the desired amount of CMAS, the feeding was halted, and the flame was maintained on the sample for the remaining 20 minute heating cycle. Following the CMAS exposure and heat treatment, the burner torch was promptly withdrawn from the sample. Subsequently, the sample was allowed to cool through ambient convection to prevent additional spallation of the coating. This cooling step facilitates the examination of the interaction between the CMAS “splats” and the TBC surface^52^.

**Fig. 24:**
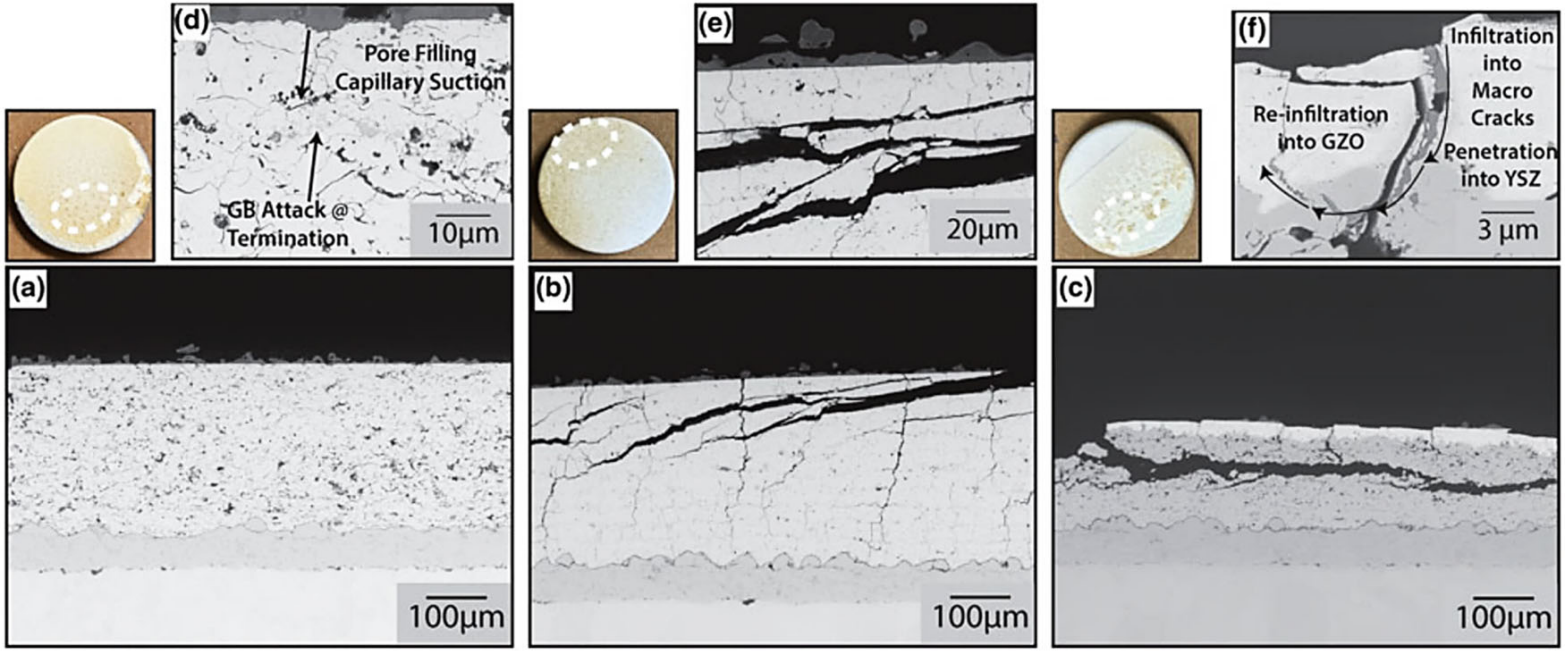
Micrograph analysis of TBCs after dynamic testing of CMAS. Cross-sectional micrographs highlighting **a** porous YSZ, **b** DVC YSZ, **c** YSZ/GZO multilayer TBCs after dynamic droplet CMAS attack in a high-loading location. **d**–**f** High-magnified micrographs of the same locations^52^.

The gradient test was carried out to examine three distinct TBCs. i.e., porous YSZ coating, dense vertical crack (DVC) coating, and multilayered gadolinium zirconate oxide (GZO)/YSZ coating. Results revealed that the porous YSZ coating displayed behavior similar to that of the static furnace exposure^73^. Despite short interval of testing with CMAS droplets, the microstructure, featuring artifacts like cavities and open pores, facilitated rapid infiltration of molten CMAS droplets due to capillary action, as discussed in section 3.2. The CMAS melt penetrated the ceramic topcoat, attacking grain boundaries, as shown in Fig. 24a. Despite the short 20 minute test cycle, no dissolution or re-precipitation occurred, and TBC delamination was not visible. In contrast, the DVC in the coating’s microstructure comprised a three-dimensional dense network with vertical macrocracks, along with a few microcracks and open cavities. However, during a brief exposure to CMAS droplets, the top surfaces of the DVC’s coating delaminated, revealing CMAS evidence within the macrocracks (refer to Fig. 24b). These findings diverged from static conditions, where infiltration occurred for DVC coatings.

In contrast to porous YSZ coating, microstructure of the DVC coating comprised a three-dimensional dense network with vertical macrocracks, along with a few microcracks and open cavities. Nonetheless, during a short exposure to CMAS droplets, the top surfaces of the DVC coating delaminated, revealing CMAS evidence within the macrocracks (see Fig. 24b). These findings are not in good agreement with the static conditions, where infiltration occurred for DVC coatings. On the other hand, the GZO/YSZ multilayered coatings mitigated the movement of molten CMAS into the coating due to reaction products that might have formed during interactions. This restriction in CMAS mobility compelled infiltration through segmented macrocracks, reaching beneath the YSZ coating (see Fig. 24c, f).

The observed results are anticipated to offer new avenues for future fundamental research endeavors, aiming at testing the TBCs against dynamic CMAS droplet interactions for longer exposure time. It is important to highlight that the coatings examined in this study were rooted in established materials and manufacturing procedures. Consequently, any advancements in these have the potential for swift translation into industrial applications.

## CMAS resistance strategies for TBCs

**Fig. 25:**
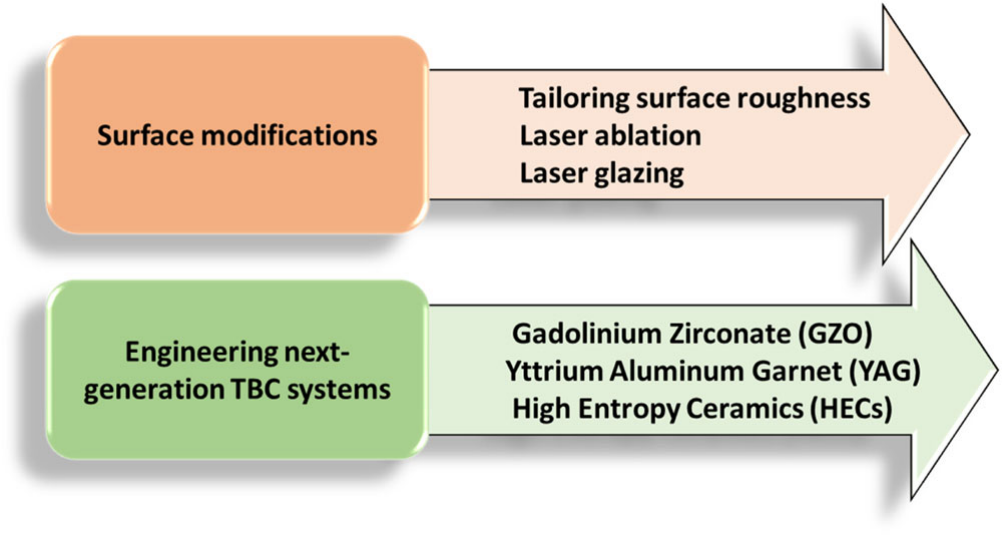
An overview of mitigation tactics for CMAS infiltration. Tactics typically consists of surface modifications via tailoring the existing TBC system and also engineering next-generation ceramic coatings to mitigate the CMAS attack.

While CMAS presents a significant threat to aeroengines, causing the degradation of TBCs through both thermomechanical and thermochemical processes, researchers have devoted their efforts to exploring innovative strategies to prevent CMAS infiltration. These efforts include modifying YSZ coatings through surface finishing and laser glazing to create surfaces resistant or repellant to CMAS. Furthermore, several researchers have conducted investigations into new ceramic coatings with the potential to mitigate CMAS effects. Figure 25 displays a concise overview of these mitigation strategies aimed at halting the infiltration of molten CMAS. Since the molten CMAS exhibits exceptional wetting properties and low viscosity, it rapidly penetrates through open pores, efficiently filling them within the ceramic topcoat. Wetting behavior greatly depends on the surface characteristics of the TBC. Nonetheless, only very few reports are available on the impact of surface characteristics (i.e., surface roughness parameters) on the wettability of melt CMAS and their spreading ability on the thermal sprayed TBCs.

**Fig. 26:**
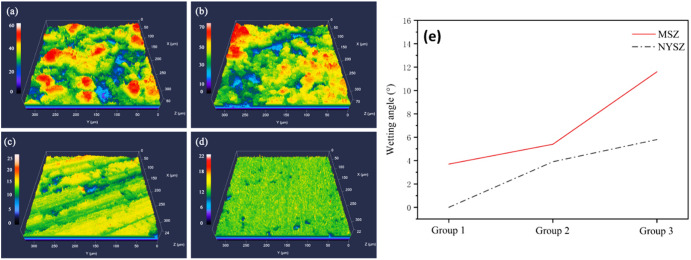
Surface morphology indicating how the surface roughness influence the wettjng behavior. Surface profilometer of the TBC coatings with different surface roughness conditions **a** NYSZ Coating in Group 1, **b**–**d** MSZ coatings in Groups 1–3. **e** shows the wetting angle between CMAS and the TBC coatings in different surface roughness conditions^74^.

Zou et al.^74^ investigated the influence of different surface roughness of nanostructured yttria-stabilized zirconia (NYSZ) and multi-component rare earth oxides modified zirconia (MSZ) APS coatings on the CMAS wettability and corrosion attack. They studied the roughness characteristics by grouping them in to three distinct characteristics. i.e., surface roughness of the group 1 displayed range between 6 and 8 µm, whilst group 2 and group 3 were grounded down to range between 1.5 and 1.8 µm, and 0.5 and 0.8 µm, respectively, as shown in Fig. 26a–d. CMAS studies were carried out using static conditions for 30 min. The effect of surface roughness was monitored according to the CMAS spreading and contact angles. A reduced spread area is likely to enhance the CMAS wetting resistance of coatings, whilst a smaller contact angle promotes greater wettability. The contact angle was measured at 0° for the NYSZ group 1 samples. In contrast, the MSZ coatings exhibited a contact angle of ~3.7°, indicating that NYSZ displays greater CMAS wettability than MSZ coating. However, it was observed that contact angles increased as the surface roughness of the coatings decreased, as illustrated in Fig. 26. This suggests that decreasing the surface roughness of both NYSZ and MSZ coatings can enhance the CMAS wetting resistance of the coatings.

**Fig. 27:**
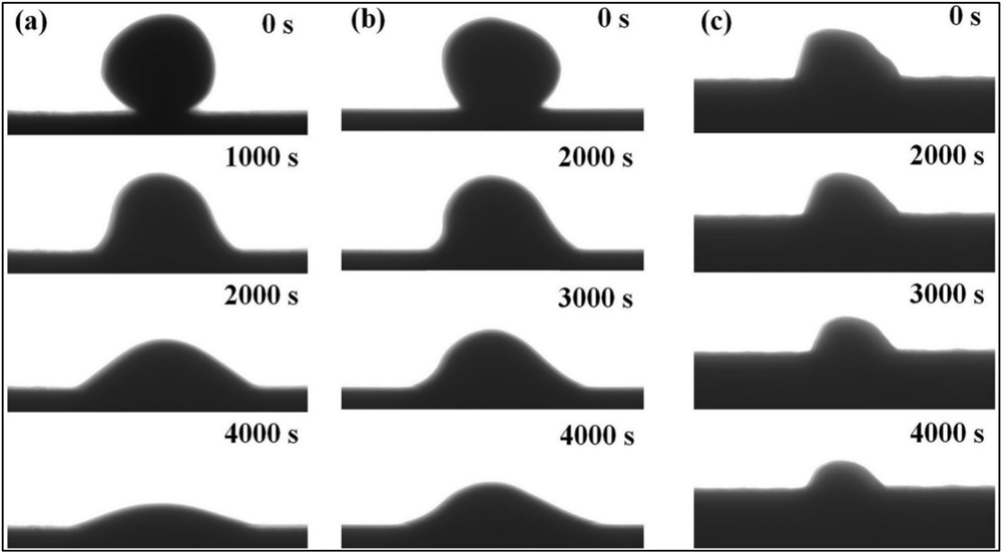
Surface morphology indicating how the surface roughness influence the wettjng behavior at different intervals. Wetting behavior of **a** as-sprayed, **b** mechanical polished, and **c** laser-ablated YSZ coatings tested at different intervals^75^.

Another study by Kang et al.^75^ investigated the wetting behavior of YSZ coating fabricated by supersonic air plasma spraying. In this regard, they utilized three different surface finish conditions, i.e., as-sprayed, mechanically polished by using standard metallographic preparation, and laser ablated using femtosecond laser, respectively. The speed of laser scanning was set as 2 mm/s under a constant average laser power of 20 mW. The as-sprayed as well as the mechanically polished YSZ coatings exhibited an average surface roughness of 6.33 µm and 1.55 µm, respectively. In contrast, the femtosecond laser-ablated coating exhibited slightly higher surface roughness of around 9.85 µm, with a typical microrod-shaped morphology because of the laser-induced phase explosion. Figure 27 illustrates the wetting processes of CMAS/YSZ coatings at 1275 °C. The initial contact angles for as-sprayed and polished coatings were 135° and 118°, respectively, with the former attributed to higher surface roughness. The laser-ablated coating had an initial angle of 85°. After 2000s, contact angles decreased to 104°, 42°, and 74° for as-sprayed, polished, and laser-ablated coatings, respectively. Subsequently, after 4000 s, final contact angles reached 56°, 28°, and 70° for the respective coatings. During the CMA/YSZ wetting process, the laser-ablated YSZ coating displayed only a slight reduction in contact angle, contact diameter, droplet height and droplet volume, highlight spreading and penetration of melt CMAS were mitigated.

Bakkar et al.^76^ also investigated the porous YSZ system by modifying the surface using laser glazing. In this regard, the laser glazing was carried out in two different configurations, by scanning the laser normal to the samples and by tilting it at 60 °C. The idea behind the laser processing is to provide a thin dense top layer of the YSZ. The study revealed fewer cracks on the surface of the porous YSZ structure after laser glazing. When exposed to CMAS attack, infiltration occurred through the cracks, but the depth of infiltration was controlled after laser glazing as compared to that seen for the as-produced porous YSZ structure. Another study by Yan et al.^77^ used pulsed Nd:YAG laser system with the incidence angle normal to the YSZ thermal sprayed coating. After laser glazing, the top surface of the YSZ coating exhibit dense columnar microstructure with a few vertical cracks. The laser glazing of ~25 µm was achieved in order to study the CMAS melt reaction. After CMAS study, the vertical cracks and open channels present in the laser-glazed surface act as a reservoir for the CMAS to infiltrate, leading to sintering and reduction in strain tolerance. Nevertheless, the glazed layer did not undergo any microstructure-induce degradation, highlighting resistance to CMAS attack. Beneath the glazed layer, delamination cracks were found, causing more damage to the TBC coating. This study highlights that reducing the density of vertical cracks by enhancing the surface quality of the laser-glazed TBC layer, perhaps can inhibit the thermomechanical and thermochemical-induce degradation.

## Engineering next-generation ceramic materials

GZO (Gd_2_Zr_2_O_7_) is one of the rare earth oxide materials that has a crystal structure crystalizes in the form of A_2_B_2_O_6_O^’^ (pyrochlore or defect fluorite). GZO exhibits immense thermal properties, which include lower thermal conductivity (~1.2–1.7 W/m K at 1000 °C)^78^ and comparable CTE (~9–11 × 10^−6^/K) compared to that of YSZ coating^20^, high-temperature durability (<1550 °C)^79^, high melting point (>2000 °C)^78^. GZO has become an potential alternate candidate for TBC materials owing to their exceptional properties. Furthermore, GZO has been studied and employed in order to counter the CMAS molten attack, with superior resistance to molten silicate deposits^39^, enable them for using as a ceramic topcoat in TBCs. Due to its larger cation size compared to 8YSZ, GZO can react with molten CMAS, forming a highly stable crystalline phase as a reaction product. This product effectively mitigates the penetration of the CMAS melt^39^.

**Fig. 28:**
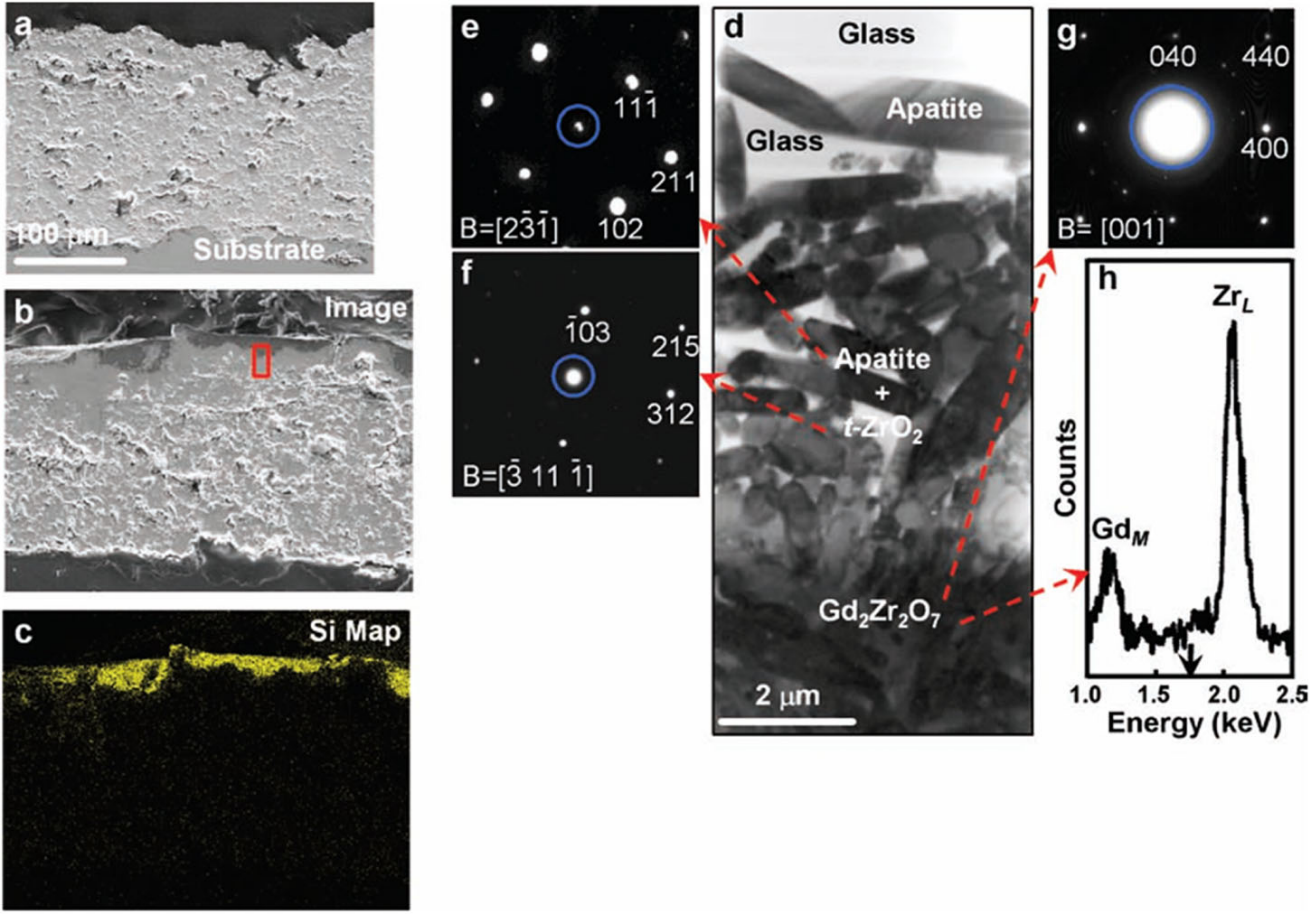
Microstructure analysis of APS deposited GZO thermal barrier coating and its response to Volcanic Ash Exposure. **a** Cross-sectional scanning electron micrographs of the APS-deposited GZO thermal barrier coating. **b**, **c** SEM and EDS map of the TBC following exposure to Eyjafjallajökull volcanic ash at 1200 °C for 24 hours in an air environment with similar magnification. **d** shows the apatite crystalline formation, and **e** indexed SAEDPs of apatite type (based on Gd_9.33_ (SiO_4_)_2_O_6_) phase and **f** *t* -ZrO_2_ tetragonal phase. **g**, **h** SAEDPs and corresponding peaks, the blue ring represents transmitted beam, and B is the zone axis^80^.

**Fig. 29:**
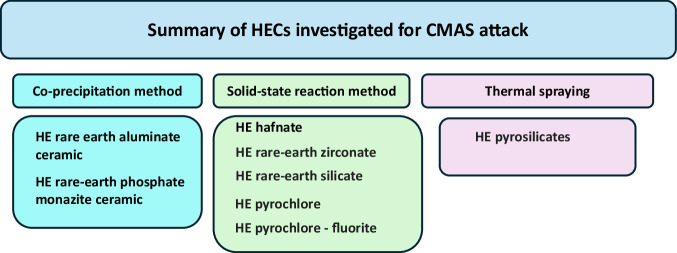
Summary providing the high-entropy ceramics (HECs) produced through different routes for CMAS studies. Investigated HECs are high entropy based rare earth aluminate, rare earth phosphate, rare earth zirconate, rare earth silicate and pyrochlore.

With the advancements of GZO, researchers investigated the effectiveness of the APS-based GZO coating as potential alternative to 8YSZ coating. For instance, in 2011, Drexler et al.^80^ conducted a groundbreaking study that examined the effects of volcanic Eyjafjallajökull ash on GZO coating produced using the APS technique. To provide a basis for comparison, the authors also produced YSZ samples and modified YSZ samples (YSZ + Al + Ti) using the same APS technique. The 24-hour heat treatment at 1200 °C was sufficient for the molten ash to attack the TBC. The investigation revealed a significant difference in the interaction phenomenon between the GZO and YSZ + Al+ Ti coatings compared to the YSZ coatings, despite their similar microstructures, each having an apparent porosity of ~20%. The infiltration depth for the GZO coating was found to be ~10 µm, as shown in Fig. 28c, whereas complete infiltration was observed in the YSZ coating after 24 hours. It should be noted that the occurrence of apatite (Gd_9.33_(SiO_4_)_2_O_6_) reaction products played a crucial role in mitigating the penetration of melt ash during interactions with the GZO coating. This was confirmed through examination of transmission electron micrographs (TEM) and the indexed SADEP pattern, see Fig. 28d, e. Interestingly, unlike the YSZ coating, no phase transition from the *t*-ZrO_2_ tetragonal phase to the *m*-ZrO_2_ monoclinic phase was observed in the GZO coating (see Fig. 29f). The emergence of apatite crystalline phase can be explained on the basis of dissolution of Gd_2_Zr_2_O_7_ grains and re-precipitation of *t*-ZrO_2_ grains. As a result, these grains interact with molten ash, leading to significant modification in the chemical composition, thereby forming apatite crystalline phase. This finding helps the researchers to understand that fabricating GZO coatings for TBCs helps resist the molten ash without any thermochemical degradation.

While the APS-based GZO coating has shown its ability to effectively combat the molten CMAS, there exhibits a few shortcomings for the GZO coating, which includes lower toughness (<1.5 MPa.√m)^81^ as well as the thermochemical incompatibilities with the alumina TGO layer^82^. It has been argued that the Gd_2_Zr_2_O_7_ tends to react with alumina to form a porous GdAlO_3_ perovskite interphase, reported previously by Leckie et al.^82^. In contrast to these shortcomings, lower CTE (8.1–10.5 × 10^−6 ^K^−1^ at 200–1000 °C) with respect to nickel-based superalloy substrate (16 × 10^−6^ K^−1^) can cause high thermal stress in-service. Such shortcomings can be alleviated with the help of bilayered or multilayered design approaches to fabricate TBC systems. For instance, Mensah et al.^83^ reported that thermal life cycling has been enhanced for the GZO/YSZ bilayer TBC system. In another study by Levi et al.^40^, GZO coatings were deposited on the top of DVC YSZ layer, which possesses higher toughness and durability. While research into bilayered/multilayered GZO TBC systems is ongoing, it is crucial to explore not only the molten CMAS attack but also comprehend the thermal and mechanical properties of these TBC systems.

In seeking next-generation TBC systems, researchers have explored the potential of YAG for mitigating the detrimental reaction of molten CMAS attack. YAG material exhibits exceptional physical and mechanical properties in comparison with YSZ for higher-temperature TBC applications. The characteristics of YAG involves high-temperature phase stability up to its melting point (1970 °C)^84^, and low thermal conductivity (0.9 W/m.K at 1300 °C)^84^. In addition to that, YAG also possess fracture toughness of 1.8 MPa.√m, lower density (4.6 g/cc) and has an excellent oxygen diffusion barrier^85^. A study conducted by Kumar et al.^84^ investigated the interaction of molten CMAS attack on solution-precursor plasma-sprayed YAG coating compared with YSZ coating. CMAS study was conducted using paste test (10 mg/cm^2^) and spritz tests, respectively. Spritz test was carried out by spraying 0.1 ml of 1 wt.% aqueous solutions of CMAS precursor chemicals on the TBC specimen before the start of every cycle. During paste test, the failure mode exhibited notable differences between the two coatings. APS YSZ coatings experienced failure in the form of flakes, with successive layers peeling off until the substrate was devoid of any coating. In contrast to YSZ coatings, YAG coatings failed as a single unit at the interface between YAG and YSZ coatings. The presence of vertical cracks in YAG coatings act as a reservoir for infiltration of molten CMAS. The study also revealed that no detrimental phase transformations have occurred during the reaction between CMAS and YAG coatings. Although, interface delamination was observed between the YAG and YSZ layers, a detail investigation related to the formation of microstructural artifacts (such as vertical cracks, pores, and channels) on the influence of CMAS infiltration on the YAG TBC needs to be investigated.

More recently, Sun et al.^86^ investigated the reactivity of CMAS on the YAG/Al2O3 eutectic samples. The samples were not produced through thermal spraying but by means of sintering. The CMAS testing was performed at 1500 °C by varying corrosion testing time (4 h, 25 h, and 100 h). The results revealed a continuous and nearly dense garnet layer was found at the interface between CMAS and Al2O3/YAG during interaction. The CMAS depth increases as function of corrosion time, with a maximum depth of 130 µm was observed after exposure to 100 h. Although thickness increased with corrosion time, the growth rate showed a decrease with the extension of reaction time, highlighting a potential candidate for utilizing TBC systems.

While zirconates have shown promise in their ability to resist CMAS attack and infiltration, significant advancements have been made in the design and production of solid solution ceramic materials through high-entropy stabilization. These materials, known as high-entropy-based ceramics (HECs)—a sub-classification of HEAs, have attracted considerable attention from researchers due to their exceptional mechanical properties in terms of their hardness and fracture toughness^87–90^, thermal stability^91^, and low thermal conductivity^91–93^. HECs consist of a minimum of five principal elements, blended in equimolar or nearly equimolar concentrations, to achieve the solid solution stabilization with high configurational entropy. In addition to the high configurational entropy, several other factors have contributed to the solid solution stabilization of thermal spray HEA coatings, as reported elsewhere^87^. Although limited, there has been a remarkable development in HECs, aiming for the replacement of state-of-the-art traditional YSZ TBC system. Thus far, various HECs have been investigated by the incorporation of rare earth elements in conjunction with high-entropy (HE) as shown in Fig. 29. To the best of author’s knowledge, only one article has been published representing the fabrication of HECs through thermal spraying for studying CMAS^94^.

The HECs includes HE rare earth monosilicates^95^, HE rare earth zirconates^96^, HE rare earth pyrochlore zirconates^97^, HE hafnate^98^, HE pyrosilicate^91^, and HE rare earth aluminate eutectic^86^. The investigation revealed these HECs as potential candidates for TBCs. A concise overview of why these HECs are considered superior candidates for TBC systems is outlined. For instance, Zhao et al.^99,100^ developed (Y_0.2_Nd_0.2_Sm_0.2_Eu_0.2_Er_0.2_)AlO_3_ and (La_0.2_Ce_0.2_Nd_0.2_Sm_0.2_Eu_0.2_)PO_4_ which displayed thermal conductivities of 4.1 W/m K and 2.08 W/m K, respectively. Another study by Zhou et al.^101^ displayed low thermal conductivity (<1 W/m K) from 300 °C to 1200 °C, higher CTE (~11 × 10^−6^ K^−1^), and good durability to thermal shock for (La_0.2_Nd_0.2_Sm_0.2_Eu_0.2_Gd_0.2_)2Zr_2_O_7_ HE rare earth zirconates prepared by APS technique. Chen et al.^95^ investigated a novel HE rare earth monosilicate (Yb_0.25_Y_0.25_Lu_0.25_Er_0.25_)_2_SiO_5_ prepared by solid state reaction method that exhibit a strong anisotropy in thermal expansion. Another study by Wright et al.^97^ reported that an ultra-low thermal conductivity and CTE closer to YSZ can be achieved by HE rare earth zirconates, highlighting the potential of using rare earth zirconates as next-generation TBC material. Although HECs exemplifies remarkable characteristics in terms of thermal stability and mechanical properties, fabrication by means of thermal spraying is still in an incubation period due to the complexity of producing the HEC feedstocks. There are a few researchers who studied the complexity of CMAS infiltration on to the HEC TBC produced by means of thermal spraying.

**Fig. 30:**
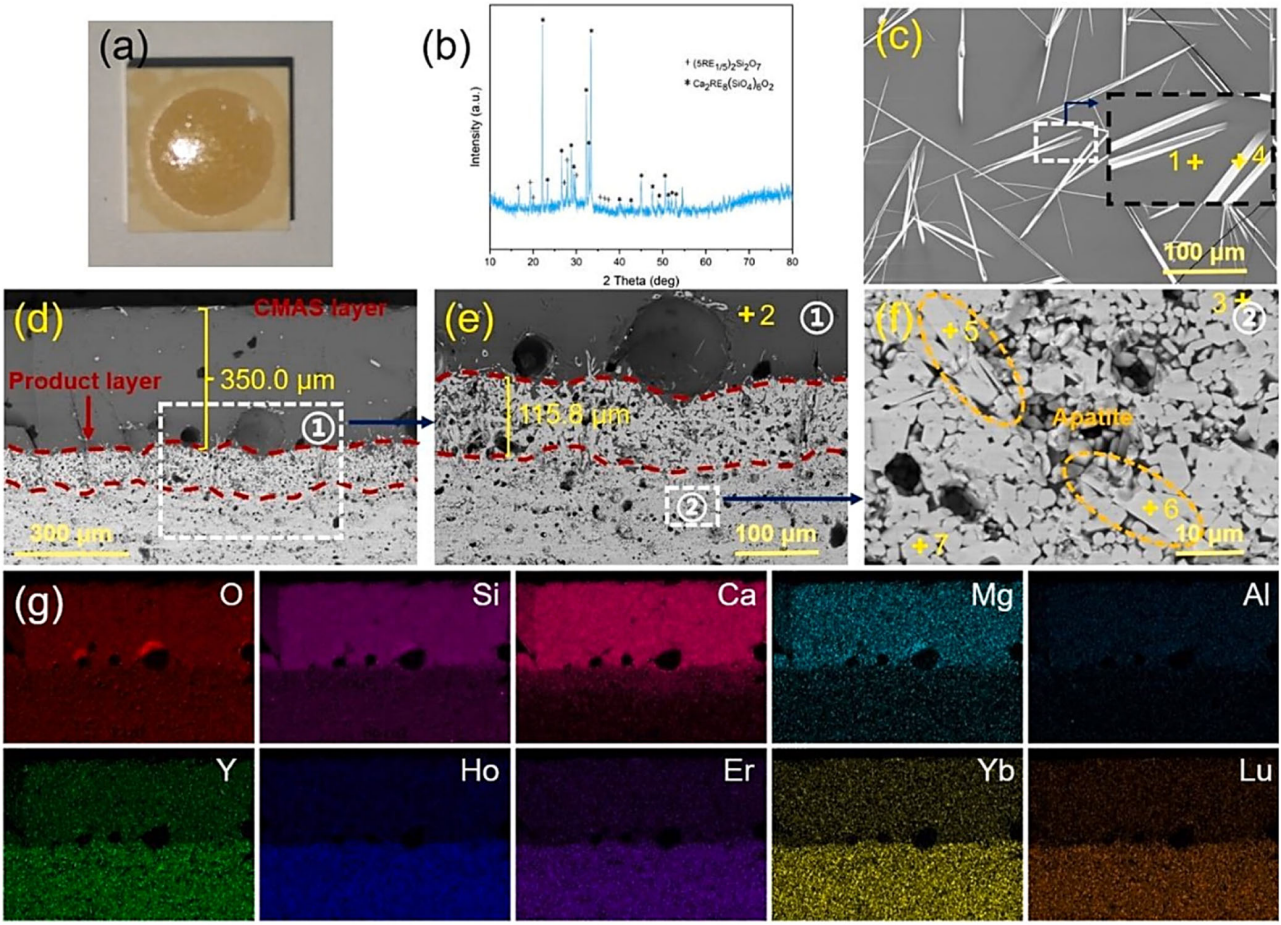
Microstructure analysis of APS deposited high-entropy pyrosilicates thermal barrier coating and its response to CMAS Exposure. Photograph of **a** CMAS on the TBC, **b** XRD pattern of the surface, **c** scanning electron microscope (SEM) image of the surface, **d**–**f** SEM images of cross-sectional views, and **g** EDS mappings illustrating the coating’s response to corrosion induced by CMAS at 1300 °C for 5 hours^94^.

More recently, Chen et al.^94^ investigated two (Yb_0.25_Lu_0.25_Ho_0.25_Er_0.25_)_2_Si_2_O_7_ and (Yb_0.2_Y_0.2_Lu_0.2_Ho_0.2_Er_0.2_)_2_Si_2_O_7_ high-entropy pyrosilicates, fabricated by means of APS technique. Static CMAS corrosion (furnace exposure) was carried out for 5 hours by setting the furnace temperature to 1300 °C. The study revealed a stable corrosion product known as apatite (Ca_2_RE_8_(SiO_4_)_6_O_2_) was formed after the reaction between the CMAS and samples. The thickness of apatite formed between CMAS and coating was ~115 µm (Fig. 30e). The apatite was in the form of long strips, with white contrast, as shown in Fig. 30c. The authors reported that the occurrence of stable corrosion product help mitigating the deep infiltration of CMAS melt (Fig. 30g). In the apatite structure, six cationic sites, each coordinated by nine oxygen ions, accommodate both RE^3+^ and Ca^2+^ ions. A more stable apatite structure is achieved when the RE^3+^ ions have a radius closer in size to that of Ca^2+^ ions. As a result, RE^3+^ ions with larger radii are more prone to precipitate from CMAS and form apatite, thereby helping mitigate the infiltration^102^. The occurrence of apatite grains, leading to reduction in CMAS infiltration for the HE rare earth zirconates and HE rare earth hafnate, also reported previously by Tu et al.^103^ and Cong et al.^98^. Thus, from the reports available on the CMAS attack on HECs it is evident that a noticeable enhancement in the CMAS resistance demonstrates that the HECs is practically a feasible next-generation TBC systems replacing the state-of-the-art YSZ system. However, studies are required to understand the different HEC systems produced by means of thermal spraying.

## Concluding remarks and future directions

This article provides a comprehensive review and understanding of the adverse effects resulting from the interaction between CMAS (calcium-magnesium-alumino silicate) and traditional thermally sprayed YSZ (yttria-stabilized zirconia) TBCs. The consequences of CMAS-induced degradation are particularly severe for aircraft, leading to damage in TBC systems through both thermomechanical and thermochemical processes.

Thermomechanical degradation manifests as high internal stress, a reduction in strain tolerance, an increase in elastic modulus, sintering, and a decrease in CTE. Simultaneously, thermochemical delamination involves infiltration through pores and open channels, phase transformation from tetragonal to monoclinic zirconia (*t*–*m* ZrO_2_), and the formation of yttria-depleted globular ZrO_2_ regions within the CMAS. These mechanisms contribute to the degradation of state-of-the-art traditional YSZ ceramic TBCs.

Numerous global studies have been conducted to explore modifications or replacements for traditional YSZ coatings. Specifically, surface tailoring through laser glazing and laser ablation have been identified as a method to increase the contact angle and reduce CMAS infiltration in YSZ coatings compared to conventional surface finishing methods. Additionally, various ceramic chemistries, ranging from zirconate compounds to multi-component perovskites, have been investigated, albeit with limited success.

Studies have demonstrated that the application of yttria aluminum garnet (YAG) and gadolinium zirconate (GZO) in different layered configurations (single, bi, and/or multilayered) enhances the durability of TBC systems by mitigating CMAS damage^73^. However, it is essential to consider the durability of the TGO (thermally grown oxide) layer when GZO reacts with the bondcoat as well as their lower toughness. Furthermore, there is a significant research gap regarding how CMAS infiltration alters the microstructure quality of the bondcoats, as well as the growth of the TGO within the TBC systems. Multilayered GZO/YSG coatings have emerged as an alternative approach, demonstrating improved CMAS resistance while meeting other requirements such as thermal stability and maintaining the integrity of the TGO layer^73^. GZO has now been in operation in aeroengines for several years. In contrast to GZO, HECs have shown promising performance in terms of low thermal conductivity, compatibility with the bondcoat, and enhanced resistance against CMAS attacks. Despite this, there is a limited body of research on the fabrication of HECs using thermal spraying technology. Among the studies reviewed in this article, some future prospectives are drawn below:

- Implementing surface modifications via laser glazing emerges as a viable and promising strategy to create CMAS-phobic surfaces on traditional YSZ TBC system. To enhance the surface quality of the laser-glazed YSZ coating layer, it is imperative to conduct parametric optimization using the design of experiment (DOE) method (for example, Box–Behnken technique)^104^. This optimization process is critical for achieving surfaces characterized by minimal vertical cracks and open channels. The occurrence of vertical cracks and network channels serves as localized sites for rapid CMAS infiltration, leading to microstructural-induced degradation. Furthermore, wetting behavior and contact angle studies are also imperative in order to understand the CMAS-phobic surfaces in conjunction with the laser-glazed surfaces. Notably, there exists a scarcity of research on laser-glazed and laser-ablated surfaces of YSZ TBC.
- Research on the dynamic interactions of CMAS is currently limited, with a focus on comprehending these interactions under active engine testing conditions and elucidating their fundamental degradation mechanisms. Studies in this area have utilized a burner test rig (oxygen-fuel flame) to subject TBC surfaces to CMAS dynamically, introducing the material either axially or radially into the flame^51^. Further investigation is necessary to explore the dynamic interaction of CMAS on CMAS-phobic surfaces, which may be created through methods such as mechanical polishing and laser surface processing. This entails spraying CMAS onto these surfaces to achieve the desired thickness and quantifying the deposited amount for a more comprehensive understanding of reaction between the CMAS and TBC systems.
- The TBC system produced via a multilayered or bilayered approach, incorporating rare earth zirconates and YSZ, has demonstrated their capability to address both intrinsic and extrinsic failure modes. The success of these approaches suggest that they have the potential to serve as viable alternatives to state-of-the-art traditional TBC system, providing further optimization in accordance with industrial standards.
- High-entropy ceramics with rare earth elements are the next-generation advanced materials for TBC systems^98,105^. Studies have shown that there is capacity to tackle the CMAS resistances with the formation of stable corrosion products (apatite). Nevertheless, there is a limited body of research focused on the thermal spraying of HECs. Notably, the OB of HECs is also lacking in order to comprehend the reactivity between CMAS and corresponding HEC systems. Therefore, further research is required to study the process-structure relationships on CMAS attack of thermal sprayed HEC TBC systems.
- Plasma spray physical vapor deposition (PS-PVD) is an intriguing technology that enables highly tailorable coatings with various rare metal elements to be processed. The PS-PVD technology bridges the gap between traditional PS-PVD techniques, which provide different combinations of tailored microstructures composed of vapor, liquid, and solid deposition units^106^. The technology is predominantly featured by a high power and long plasma plume of ~2000 mm, with a low-pressure working environment. Thus, the ceramic materials can easily be vaporized during the spraying, allowing the presence of gas, liquid, and solid particles in the plume. To the best of author’s knowledge, there is only one article presenting the use of PS-PVD for encountering the CMAS attack^107^. Owing to the tailored quasi-columnar structure, the CMAS penetration was lowered, enabling them as a potential thermal spraying technique. Further research is necessary in order to understand the feasibility of using PS-PVD with tailored microstructure for combating CMAS molten attack.

## Data Availability

The authors confirm that the data supporting the findings of this study are available within the article.
